# Application of the Meerwein reaction of 1,4-benzoquinone to a metal-free synthesis of benzofuropyridine analogues

**DOI:** 10.3762/bjoc.17.79

**Published:** 2021-04-30

**Authors:** Rashmi Singh, Tomas Horsten, Rashmi Prakash, Swapan Dey, Wim Dehaen

**Affiliations:** 1 Department of Chemistry, Indian Institute of Technology (Indian School of Mines) Dhanbad, Dhanbad 826004, India; 2 Department of Molecular Design and Synthesis, Department of Chemistry, KU Leuven, Celestijnenlaan 200F, 3001 Leuven, Belgium

**Keywords:** benzofuropyridines, benzoquinones, dibenzofurans, Meerwein reaction, metal-free synthesis

## Abstract

Several new heterocyclic systems based on a hydroxybenzofuro[2,3-*b*]pyridine building block were prepared. This benzofuropyridine is easily available from the Meerwein reaction of benzoquinone and a heterocyclic diazonium salt, followed by reduction and cyclization. Electrophilic substitution and further condensations give polycyclic systems, including oxazolo- and chromeno-fused analogues.

## Introduction

**Figure 1:**
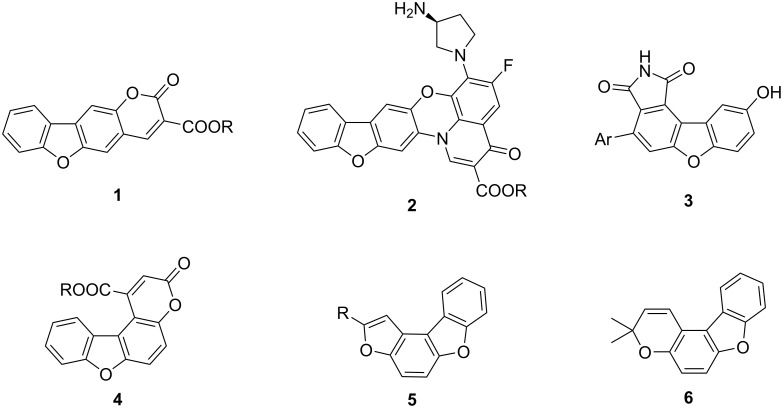
Biologically relevant 2-oxydibenzofuran-containing structures **1**–**6**.

Dibenzofurans are important oxygen-containing heterocycles present in multiple natural products [1–2] and have broad applications in areas ranging from medicinal chemistry [1–10] to materials science [11]. Figure 1 presents a few examples of dibenzofuran-containing molecules. Benzofurocoumarin analogues of **1** have antiproliferative effects on human cancer cell lines [12–13]. Fluoroquinophenoxazines **2** have been used as telomerase inhibitors in anticancer research [14]. Furthermore, benzofuroisoindoles **3** were part of a kinase inhibitor study [15]. Photobiologically active psoralene (linear furocoumarin) dibenzofurans **1** and angular furanocoumarin dibenzofurans **4** were also explored for the treatment of various skin diseases [16]. Furthermore, 2-substituted benzofurobenzofurans **5** with antitubercular activity have been reported [17]. Prado et al. reported the antimycobacterial activity of furo[3,2-*f*]chromene analogue **6** [18].

**Figure 2:**
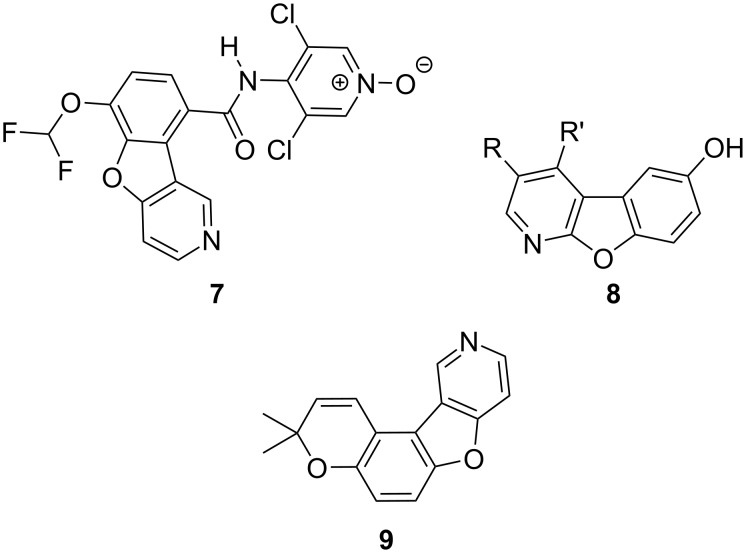
Representative bioactive structures containing benzofuro-fused pyridine analogues **7**–**9**.

The aza analogues of dibenzofuran have been less explored, although they may have significant bioactivity. Introducing nitrogen to the dibenzofuran system is expected to increase the water solubility and potential bioavailability due to enhanced hydrogen bonding. Figure 2 presents a few examples of azadibenzofuran molecules. One example of a biologically active benzofuropyridine is revamilast (**7**), which has been used in Phase II clinical trials, studying the treatment of asthma and rheumatoid arthritis [19]. Other examples are the hydroxybenzofuro[2,3-*b*]pyridines **8** with efflux pump inhibitory activity useful in chemotherapy [20–21]. These compounds are presumably multitargeting drugs because of the diverse applications as insulin-like growth factor 1 receptor (IGF-1R) inhibitors [22], selective GSK-3β inhibitors important in Alzheimer's disease [23–24], and cyclin-dependent kinase (CDK) inhibitors [25–27]. Lastly, the aza analogue **9** of previously reported furo[3,2-*f*]chromene **6** was examined for antimycobacterial activity [28].

This evidence encouraged us to investigate new methodologies for the synthesis of aza analogues of dibenzofuran from commercially available aminochloropyridines. Furthermore, a diverse set of polycyclic derivatives was designed. The procedures towards such polycyclic building blocks include C–H-arylation strategies. In the classical Meerwein reaction, aryldiazonium salts are used as the reagents to couple aryl groups to electron-poor alkenes, and this process is assumed to proceed via a free-radical mechanism [29]. Similar reaction intermediates can be prepared using precursors such as organoboron reagents [30]. However, due to the accessibility, aryldiazonium salts are the reagents of choice. They can be prepared starting from the commercially available corresponding anilines. The present work focuses on a metal-free approach for the synthesis of benzofuropyridine analogues.

## Results and Discussion

**Scheme 1:**
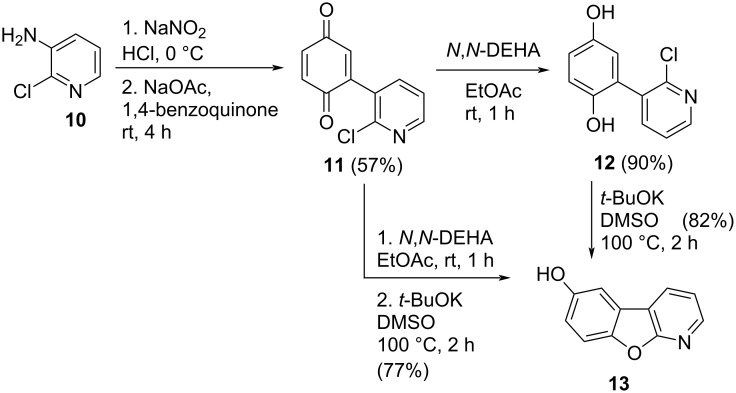
Strategy for metal-free access to benzofuropyridine **13**.

The synthesis of target compound **13** involved three steps (Scheme 1). C–H Arylation, as needed in the first step, is usually carried out using transition metal catalysis [31]. Furthermore, various metal-based approaches for arylation of quinone involving electrochemical [32], oxidative [33], and photochemical methods [34–36] are also available in the literature [37]. Langer and co-workers reported the synthesis of benzofuropyridines based on a domino reaction of 3-chlorochromones with aminoheterocycles [38]. Alternatively, the classical Meerwein reaction can be applied starting from 3-amino-2-chloropyridine (**10**), which was transformed into the diazonium salt and coupled in situ with 1,4-benzoquinone, forming arylated quinone **11** without an additional reducing agent [39]. The quinone was reduced to hydroquinone **12** with *N*,*N-*diethylhydroxylamine (*N*,*N*-DEHA) and cyclized via intramolecular nucleophilic aromatic substitution to isolate 6-hydroxybenzofuro[2,3-*b*]pyridine (**13**) with 82% yield. Conveniently, the synthesis of **13** was achieved in a one-pot reaction from **11** with no significant differences in the yield. To the best of our knowledge, this is the first procedure toward compound **13**, without additional substituents on the pyridine ring. Furthermore, this method is complementary to the most common routes towards the biologically active 1-aza-9-oxafluorenes [20–26].

**Scheme 2:**
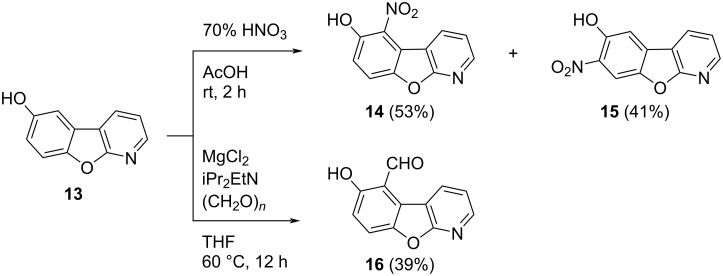
Electrophilic aromatic substitution of 6-hydroxybenzofuro[2,3-*b*]pyridine (**13**).

To expand the library of derivatives containing core structure **13**, electrophilic aromatic substitution of this compound was explored (Scheme 2). Nitration of **13** using 70% nitric acid in glacial acetic acid gave the corresponding regioisomers **14** and **15** in 53% and 41% isolated yield, respectively. The ^1^H NMR spectrum of **15** showed two apparent singlets separately at δ_H_ 7.86 and δ_H_ 8.35, which confirmed that the hydrogen atoms of the phenol ring had a *para*-relationship. The ^1^H NMR spectrum of compound **14** was similar to that of compound **15** concerning the pyridine part. However, two separate doublet signals appeared at δ_H_ 7.98 (d, *J* = 9.1 Hz, 1H) and δ_H_ 7.35 (d, *J* = 9.0 Hz, 1H) indicating an *ortho*-relationship between the hydrogen atoms positioned on the phenolic ring.

Several methods of formylation of **13** were attempted, e.g., Vilsmeier formylation, where only the unstable formate ester was formed. Following the Duff formylation procedure, only traces of aldehyde **16** were detected. Rieche formylation with either SnCl_4_ or TiCl_4_ resulted in a low conversion of the starting material and only traces of **16** due to the limited solubility of **13** in DCM, DCE, or chloroform. Furthermore, **16** was isolated after a Reimer–Tiemann formylation; however, only in 13% yield. Finally, Casnati–Skattebøl formylation of **13** using MgCl_2_, iPr_2_EtN, and paraformaldehyde, i.e., (CH_2_O)*_n_*, has been successful in regioselectively affording aldehyde **16** in a reasonable yield of 39%. The regioselectivity of compound **16** was confirmed by ^1^H NMR spectroscopy, which indicated the presence of two doublets at δ_H_ 7.88 (d, *J* = 9 Hz, 1H) and δ_H_ 7.19 (d, *J* = 8.9 Hz, 1H), corresponding to the two adjacent hydrogen atoms of the phenolic ring. One singlet was observed at δ_H_ 10.58, evidencing the aldehyde proton. This access to previously unknown compounds **14**–**16** opens many synthetic possibilities for the preparation of novel fused derivatives of 1-aza-9-oxafluorene.

**Scheme 3:**
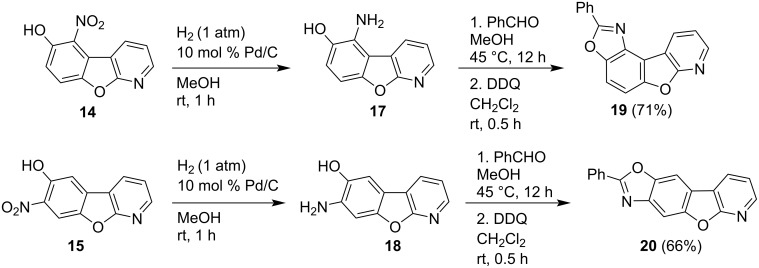
Synthesis of isomeric oxazole-fused derivatives.

The nitro compounds **14** and **15** were reduced to the corresponding aniline derivatives using hydrogen and Pd/C as a catalyst. The resulting aminophenols **17** and **18** were further converted to novel oxazole-fused derivatives **19** and **20**, respectively, by condensation with benzaldehyde and subsequent 2,3-dichloro-5.6-dicyano-*p*-benzoquinone (DDQ)-mediated oxidation (Scheme 3).

**Scheme 4:**
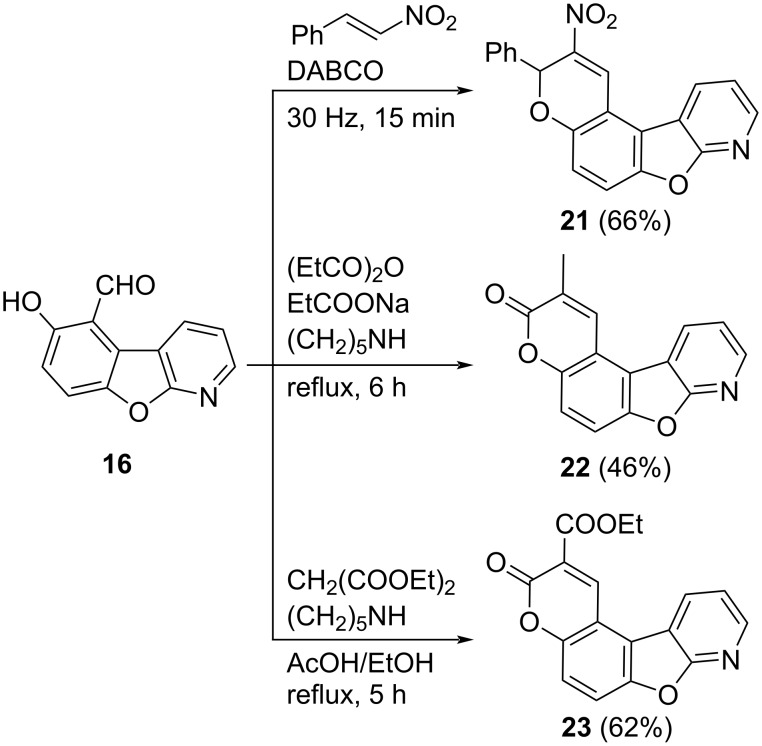
Fused derivatives from **16**.

Aldehyde building block **16** was a versatile starting material for further cyclization reactions. Synthesis of nitrochromenobenzofuropyridine **21** was achieved after treatment of **16** with nitrostyrene in the presence of DABCO applying our previously reported ball milling procedure [40]. The synthesis of **22** was performed using the Perkin reaction [41]. The reaction of **16** with propionic anhydride and the corresponding sodium salt in the presence of a catalytic amount of piperidine afforded pyridopsoralen **22** in 46% yield. Analogously, pyridopsoralen **23** was prepared from **16** by Knoevenagel condensation with diethyl malonate and subsequent lactonization with 62% yield (Scheme 4) [13]. To the best of our understanding, the scaffolds **21**–**23** are novel and may have a potential medicinal interest.

## Conclusion

In conclusion, we have successfully synthesized hydroxy-substituted pyridobenzofuran **13**. Furthermore, nitration of **13** yielded two regioisomers, **14** and **15**, which were further converted to oxazoles **19** and **20**. Formylation of **13** was regioselective in forming **16**, which is a valuable building block for various condensation reactions to yield a diverse set of products, such as polycyclic fused nitrochromenes **21** as well as pyridopsoralens **22** and **23**. All these novel scaffolds are interesting structures with potential medicinal applications, and we plan to expand this chemistry and carry out a bioactivity study in due course.

## Supporting Information

File 1 Experimental part as well as ^1^H and ^13^C NMR data.
