# Supplementary material for: Application of the Meerwein reaction of 1,4-benzoquinone to a metal-free synthesis of benzofuropyridine analogues
Source: Beilstein J Org Chem. 2021 Apr 30;17:977–82. doi: 10.3762/bjoc.17.79 (PMC8093549; doi:10.3762/bjoc.17.79)

BEILSTEIN JOURNAL OF ORGANIC CHEMISTRY

Supporting Information

for

Application of the Meerwein reaction of 1,4-benzoquinone to a metal-free synthesis of benzofuropyridine analogues

Rashmi Singh, Tomas Horsten, Rashmi Prakash, Swapan Dey and Wim Dehaen

Beilstein J. Org. Chem. **2021,** *17,* 977–982. doi:10.3762/bjoc.17.79

Experimental part as well as ^{1}H and ^{13}C NMR data

License and Terms: This is a supporting information file under the terms of the Creative Commons Attribution License (https://creativecommons.org/licenses/by/4.0). Please note that the reuse, redistribution and reproduction in particular requires that the author(s) and source are credited and that individual graphics may be subject to special legal provisions.
The license is subject to the *Beilstein Journal of Organic Chemistry* terms and conditions: (https://www.beilstein-journals.org/bjoc/terms)

Experimental

General: Chemicals received from commercial sources (Sigma-Aldrich, Acros Organics, J & K Scientific, Alfa Aesar, or TCI Chemicals) were used without further purification. Column chromatography was performed over Acros silica gel (60 Å). Dry reaction solvents were purchased from commercial sources. Thin-layer chromatography (TLC) was performed on silica gel 0.20 mm 60 with fluorescent indicator UV254 (precoated aluminum sheets) from Merck. For column chromatography, 60–200 mesh silica gel 60 (Acros) was used as a stationary phase. NMR spectra were acquired on commercial instruments (Bruker Avance 300 MHz, Bruker AMX 400 MHz or Bruker Avance II^+ 600 MHz) and chemical shifts (δ) are reported in parts per million (ppm), referenced to tetramethylsilane (^{1}H) or the internal NMR solvent signal (^{13}C). High-resolution mass spectra were acquired on a quadrupole orthogonal acceleration time-of-flight mass spectrometer (Synapt G2 HDMS, Waters, Milford, MA, USA). Samples were infused at 3μL/min, and spectra were obtained in positive mode with a resolution of 15 000 (FWHM) using leucine enkephalin as lock mass. Melting points (uncorrected) were determined using a Reichert Thermovar apparatus. Ball milling experiments were performed on a Retsch mixer mill MM 400.

2-(2-Chloropyridin-3-yl)cyclohexa-2,5-diene-1,4-dione (11). A mixture of 3-amino-2-chloropyridine (**10**, 0.257 g, 2 mmol) in H_2O (2 mL) and HCl (37%, 1 mL) was cooled down to 5 °C, after which a solution of $NaNO_2$ (0.166 g, 2.4 mmol) in H_2O (1 mL) was added dropwise whilst maintaining the temperature below 5 °C. In a second flask, a solution of 1,4-benzoquinone (0.864 g, 8 mmol) and NaOAc (0.443 g, 5.4 mmol) in H_2O (20 mL) was prepared and kept at 10 °C. Finally, the diazonium salt was added gradually to this solution and stirred for another 4 h at room temperature. Compound **11** was obtained (250 mg, 1.14 mmol, 57%) as a yellow solid after filtration and column chromatography (18% v/v EtOAc in petroleum ether). The reaction was easily scaled up, leading to a gram-scale syntheses (15.55 mmol) without significant loss in the yield, i.e., 53%. **Mp** 127-129 °C; **^{1}H NMR** (400 MHz, $CDCl_3$) *δ*: 8.47 (dd, *J* = 4.8, 2 Hz, 1H), 7.61 (dd, *J* = 7.6, 2 Hz, 1H), 7.36 (dd, *J* = 7.6, 4.8 Hz, 1H), 6.95 – 6.87 (m, 2H), 6.86 (d, *J* = 2.3 Hz, 1H). **^{13}C NMR** (101 MHz, $CDCl_3$) *δ*: 186.74, 184.58, 150.31, 149.71, 143.98, 139.46, 136.70, 136.63, 135.40, 128.86, 122.24. **HRMS** (ESI+): *m/z* calculated for $C_{11}H_6ClNO_2$ $[M+H]^+$: 220.0160, found 220.0147.

2-(2-Chloropyridin-3-yl)cyclohexa-2,5-diene-1,4-diol (12). To a solution of **11** (0.5 g, 2.28 mmol) in EtOAc (5 mL), *N*,*N*-diethylhydroxylamine (450 µL, 4.55 mmol) was added at room temperature. After 1 h, the reaction mixture was quenched in HCl (4%) and extracted with EtOAc. The combined organic layers were washed with brine, dried over $MgSO_4$ and finally, the solvent was removed under reduced pressure. After column chromatography (50% v/v EtOAc in petroleum ether), the hydroquinone **12** was obtained as a yellowish solid (455 mg, 90%). **Mp** 212 – 216 °C; **^{1}H NMR** (400 MHz, DMSO-d_6) δ: 8.89 (s, 1H), 8.87 (s, 1H), 8.36 (dd, J = 4.8, 2 Hz, 1H), 7.80 (dd, J = 7.5, 2 Hz, 1H), 7.44 (dd, J = 7.5, 5 Hz, 1H), 6.80 (d, J = 8.64 Hz, 1H), 6.66 (dd, J = 8.7 Hz, 3 Hz, 1H), 6.53 (d, J = 3 Hz, 1H). **^{13}C NMR** (101 MHz, DMSO-d_6) δ: 149.75, 149.55, 148.20, 146.90, 140.84, 134.25, 124.65, 122.81, 116.69, 116.39, 116.28. **HRMS** (ESI+): *m/z* calculated for $C_{11}H_8ClNO_2$ $[M+H]^+$: 222.0244, found 222.0305.

Benzofuro[2,3-*b*]pyridin-6-ol (13). The reduced compound **12** (0.345 g, 1.56 mmol) was brought together with potassium *tert*-butoxide (0.524 g, 4.7 mmol) in DMSO (3.5 mL) and heated at 100 °C for 2 h. The mixture was cooled down, poured into ice-cooled water and extracted with EtOAc. The combined organic layers were washed with brine, dried over $MgSO_4$ and finally, the solvent was removed under reduced pressure. After flash column chromatography (30% v/v EtOAc in petroleum ether), **13** was obtained as an off-white solid. (236 mg, 82%). **Mp** 230-232 °C; **^{1}H NMR** (400 MHz, DMSO-d_6) δ: 9.58 (s, 1H), 8.50 (dd, J = 7.6, 1.8 Hz, 1H), 8.39 (dd, J = 4.9, 1.7 Hz, 1H), 7.54 (d, J = 8.8 Hz, 1H), 7.50 (d, J = 2.5 Hz, 1H), 7.40 (dd, J = 7.6, 4.9 Hz, 1H), 7.02 (dd, J = 8.8, 2.6 Hz, 1H). **^{13}C NMR** (100 MHz, DMSO-d_6) δ: 163.03, 153.88, 147.44, 146.17, 130.64, 122.64, 119.25, 116.80, 116.57, 112.29, 106.87. **HRMS** (ESI+): *m/z* calculated for $C_{11}H_7NO_2$ $[M+H]^+$: 186.0477, found 186.0528.

One-pot synthesis of benzofuro[2,3-*b*]pyridin-6-ol (13). To a solution of **11** (0.5 g, 2.28 mmol) in EtOAc (5 mL), *N*,*N*-diethylhydroxylamine (450 µL, 4.55 mmol) was added at room temperature. After 1 h, the solvent was removed under reduced pressure. The reduced compound (0.874 g, 4.19 mmol) was then brought together with potassium *tert*-butoxide (1.4 g, 12.57 mmol) in DMSO (9 mL) and heated for 2 h at 100 °C. After this period, the mixture was cooled, poured into water, and extracted with EtOAc. The combined organic layers were washed with brine, dried over $MgSO_4$ and finally, the solvent was removed under reduced pressure. After flash column chromatography (30% v/v EtOAc in petroleum ether), the product

was obtained as a yellow solid identical to the sample obtained earlier (325 mg, 77% over two steps).

5-Nitrobenzofuro[2,3-*b*]pyridin-6-ol (14) and 7-nitrobenzofuro[2,3-*b*]pyridin-6-ol (15). Nitric acid (70%, 60 µL 1.35 mmol) was added to a stirred solution of compound **13** (0.1 g, 0.54 mmol) in glacial acetic acid (3mL) at 0 °C. After 10 min, the reaction mixture was further stirred at room temperature for 2 h. The reaction was poured over ice and extracted with EtOAc (3 × 50 mL). The combined organic layers were washed with brine, dried over $MgSO_4$, and the solvent was evaporated under reduced pressure. Purification by column chromatography (12% v/v EtOAc in petroleum ether) afforded the two regioisomers **14** and **15** as yellow solids.

Regioisomer 14 Yellow solid (66 mg, 53%)**. Mp** 214-216 °C; **^{1}H NMR** (400 MHz, DMSO-*d*$_6$) *δ*: 11.20 (s, 1H), 8.54 (dd, *J* = 4.9, 1.7 Hz, 1H), 8.37 (dd, *J* = 7.9, 1.7 Hz, 1H), 7.98 (d *J* = 9.1, 1H), 7.52 (dd, *J* = 7.88, 4.8 Hz, 1H), 7.35 (d, *J* = 9.0 Hz, 1H). **^{13}C NMR** (101 MHz, DMSO-*d*$_6$) *δ*: 162.69, 148.61, 148.09, 146.38, 133.07, 130.57, 120.07, 119.17, 118.15, 115.86, 113.33. **HRMS** (ESI+): *m/z* calculated for $C_{11}H_6N_2O_4$ [M+H]$^+$: 231.0328, found 231.0378.

Regioisomer 15. Yellow solid (51 mg, 41%). **Mp** 222-224 °C; **^{1}H NMR** (400 MHz, DMSO-*d*$_6$) *δ*: 10.97 (s, 1H), 8.72 (dd, *J* = 7.7, 1.8 Hz, 1H), 8.54 (dd, *J* = 4.9, 1.8 Hz, 1H), 8.35 (s, 1H), 7.86 (s, 1H), 7.54 (dd, *J* = 7.7, 4.9 Hz, 1H). **^{13}C NMR** (101 MHz, DMSO-*d*$_6$) *δ*: 164.26, 148.68, 148.59, 145.50, 136.80, 132.76, 128.01, 120.17, 115.02, 110.64, 108.45. **HRMS** (ESI+): *m/z* calculated for $C_{11}H_6N_2O_4$ [M+H] $^+$: 231.0328, found 231.0404.

6-Hydroxybenzofuro[2,3-*b*]pyridine-5-carbaldehyde (16). The reaction was performed with the rigorous exclusion of moisture and air. Diisopropylethylamine was distilled over CaH_2 and stored over molecular sieves under argon. Paraformaldehyde, anhydrous $MgCl_2$, and **13** were dried overnight over P_2O_5 at a high-vacuum. To a screw-capped reaction tube equipped with a magnetic stirring bar, **13** (92 mg, 0.5 mmol), $MgCl_2$ (475 mg, 5 mmol) and iPr_2EtN (387 mg, 3 mmol), 5 mL of anhydrous degassed THF was added. The reaction was purged with argon while applying sonication. After 15 min, paraformaldehyde (300 mg, 10 mmol) was added and

the reaction was flushed with argon and closed tightly. The reaction was stirred vigorously at 60 °C for 12 h. Afterwards, the reaction was cooled to room temperature and 5 mL of a 1 M HCl solution in water was added followed by stirring at room temperature for 30 min. The solution was diluted with 100 mL water and extracted with ethyl acetate (3 × 100 mL). Combined organic layers were washed with brine and dried over Na_2SO_4. The solvent was evaporated under reduced pressure. The crude reaction mixture was purified by column chromatography (15% v/v EtOAc in petroleum ether) to obtain **16** as a yellow solid (42 mg, 39%). **Mp** 238-240 °C; **^{1}H NMR** (400 MHz, DMSO-*d*$_6$) *δ*: 10.58 (s,1H), 9.12 (dd, *J* = 7.9, 1.8 Hz, 1H), 8.45 (dd, *J* = 4.8, 1.8 Hz, 1H), 7.88 (d, *J* = 9.0 Hz, 1H), 7.43 (dd, *J* = 7.8, 4.9 Hz, 1H), 7.19 (d, *J* = 8.9 Hz, 1H).**^{13}C NMR** (101 MHz, DMSO-*d*$_6$) *δ*: 190.33, 163.11, 159.30, 147.40, 147.22, 135.43, 120.04, 119.85, 119.43, 117.93, 117.23, 115.77.**HRMS** (ESI+): *m/z* calculated for $C_{12}H_7NO_3$ $[M+H]^+$: 214.0426, found 214.0478.

5-Aminobenzofuro[2,3-*b*]pyridin-6-ol (17). A solution of **14** (115 mg, 0.50 mmol) and 10% palladium on carbon (5.32 mg, 0.05 mmol) in MeOH (3 mL) was placed in a round-bottom flask under hydrogen atmosphere at room temperature. The mixture was stirred until the reaction was finished according to TLC analysis (approximately 1 h). The mixture was filtered through Celite®, and the filtrate was evaporated. The residue was passed through a silica plug and evaporated to give **17** as a red oil (87 mg, 87%), which was used immediately in the following reaction. **^{1}H NMR** (400 MHz, DMSO-*d*$_6$) *δ*: 9.21 (s, 1H), 8.66 (dd, *J* = 7.6, 1.7 Hz, 1H), 8.27 (dd, *J* = 4.9, 1.7 Hz, 1H), 7.37 (dd, *J* = 7.6, 5.0 Hz, 1H), 6.93 (d, *J* = 8.5 Hz, 1H), 6.75 (d, *J* = 8.4 Hz, 1H). **^{13}C NMR** (101 MHz, DMSO-*d*$_6$) *δ*: 162.22, 148.18, 143.89, 139.36, 131.84, 130.32, 118.69, 116.91, 115.06, 108.53, 97.84. **HRMS** (ESI+): *m/z* calculated for $C_{11}H_8N_2O_2$ $[M+H]^+$: 201.0586, found 201.0644.

7-Aminobenzofuro[2,3-*b*]pyridin-6-ol (18). A solution of **15** (115 mg, 0.50 mmol) and 10% palladium on carbon (5.32 mg, 0.05 mmol) in MeOH (3 mL) was placed in a round-bottom flask under hydrogen atmosphere at room temperature. The mixture was stirred until the reaction was finished according to TLC analysis (approximately 1 h). The mixture was filtered through Celite®, and the filtrate was evaporated. The residue was passed through a silica plug and evaporated to give **18** as a red oil (83 mg, 83%), which was used immediately in the following reaction. **^{1}H NMR** (400 MHz, DMSO-*d*$_6$) *δ*: 9.37 (s, 1H), 8.18 (dd, *J* = 7.7, 2 Hz,

1H), 8.13 (dd, *J* = 4.92, 1.7 Hz, 1H) 7.26 (dd, *J* = 7.5, 5.0 Hz, 1H), 7.24 (s, 1H), 6.85 (s, 1H). **^{13}C NMR** (100 MHz, DMSO-*d*$_6$) *δ*: 162.15, 149.44, 142.26, 141.38, 139.87, 127.36, 118.88, 117.90, 109.22, 105.19, 95.30. **HRMS** (ESI+): *m/z* calculated for $C_{11}H_8N_2O_2$ [M+H] $^+$: 201.0586, found 201.0657.

2-Phenyloxazolo[4',5':4,5]benzofuro[2,3-*b*]pyridine (19). This was carried out in a manner analogous to a known literature procedure [1]. To a solution of **17** (0.109 g, 0.54 mmol) in MeOH (5 mL) was added benzaldehyde (110 μL, 1.08 mmol). The resulting mixture was heated at 45 °C for 12 h. After concentration under reduced pressure, the residue was dissolved in CH_2Cl_2 (10 mL), and DDQ (0.245 g, 1.08 mmol) was added. The reaction mixture was stirred at room temperature for 30 min, the resulting mixture was diluted with additional CH_2Cl_2 (10 mL) and washed sequentially with saturated $NaHCO_3$ (2 × 10 mL) and brine (10 mL). The organic layer was dried over anhydrous $MgSO_4$. The solvent was evaporated to dryness. Purification by column chromatography (12% v/v EtOAc in petroleum ether) afforded **19** as a white solid (110 mg, 0.383 mmol, 71%). **Mp** 208-210 °C; **^{1}H NMR** (400 MHz, DMSO-*d*$_6$) *δ*: 8.66 (dd, *J* = 7.6, 1.7 Hz, 1H), 8.54 (dd, *J* = 4.9, 1.8 Hz, 1H), 8.31 – 8.26 (m, 2H), 8.02 (d, *J* = 9.0 Hz, 1H), 7.83 (d, *J* = 9.0 Hz, 1H), 7.69 – 7.63 (m, 3H), 7.60 (dd, *J* = 7.6, 5 Hz, 1H). **^{13}C NMR** (100 MHz, DMSO-*d*$_6$) *δ*: 164.18, 162.73, 151.51, 147.22, 146.78, 135.93, 132.31, 131.53, 129.46, 127.49, 126.26, 120.31, 114.72, 113.01, 110.83, 109.16. **HRMS** (ESI+): *m/z* calculated for $C_{18}H_{10}N_2O_2$ [M+H]$^+$: 287.0742, found 287.0798.

2-Phenyloxazolo[5',4':5,6]benzofuro[2,3-*b*]pyridine (20). In a manner analogous to a procedure from Reference [1]. To a solution of **18** (0.100 g, 0.5 mmol) in MeOH (5 mL) was added benzaldehyde (102 μL, 1.0 mmol). The resulting mixture was heated at 45 °C for 12 h. After concentration under reduced pressure, the residue was dissolved in CH_2Cl_2 (10 mL), and DDQ (0.250 g, 1.1 mmol) was added. The reaction mixture was stirred at room temperature for 30 min, the resulting mixture was diluted with additional CH_2Cl_2 (10 mL) and washed sequentially with saturated $NaHCO_3$ (2 × 10 mL) and brine (10 mL). The organic layer was dried over anhydrous $MgSO_4$. The solvent was evaporated to dryness. Purification by column chromatography (12% v/v EtOAc in petroleum ether) afforded **20** as a white solid (94 mg, 66%). **Mp** 268-270 °C; **^{1}H NMR** (400 MHz, $CDCl_3$) *δ*: 8.49 (dd, *J* = 4.9, 1.7 Hz, 1H), 8.34 – 8.28 (m, 3H), 8.10 (s, 1H), 8.00 (s, 1H), 7.59 – 7.54 (m, 3H), 7.39 (dd, *J* = 7.6, 4.9 Hz, 1H).

^{13}C NMR (100 MHz, $CDCl_3$) *δ*: 164.88, 164.01, 152.25, 147.73, 146.66, 143.00, 132.09, 129.64, 129.18, 127.94, 127.02, 120.57, 119.28, 117.29, 103.15, 102.30. **HRMS** (ESI+): *m/z* calculated for $C_{18}H_{10}N_2O_2$ $[M+H]^+$: 287.0742, found 287.0820.

2-Nitro-3-phenyl-3*H*-chromeno[5',6':4,5]furo[2,3-*b*]pyridine (21). To a grinding jar equipped with one grinding ball, **16** (0.106 g, 0.5 mmol), β-nitrostyrene (0.074 g, 0.5 mmol) and DABCO (6 mg, 0.05 mmol) were added subsequently. Mixing was performed at 30 Hz in three consecutive cycles of 15 minutes until full conversion was indicated by TLC analysis [2]. The reaction mixture was purified by column chromatography (18% v/v EtOAc in petroleum ether) to yield **21** as a yellow solid. (113 mg, 66%) **Mp** >300 °C; **^{1}H NMR** (400 MHz, $CDCl_3$) *δ*: 8.62 (s, 1H), 8.54 (dd, *J* = 4.9, 1.7 Hz, 1H), 8.48 (dd, *J* = 7.7, 1.7 Hz, 1H), 7.59 (d, *J* = 8.9 Hz, 1H), 7.47 – 7.39 (m, 3H), 7.35 – 7.29 (m, 3H), 7.07 (d, 1H), 6.71 (s, 1H). **^{13}C NMR** (150 MHz, $CDCl_3$) *δ*: 163.82, 150.29, 149.92, 147.80, 142.03, 136.24, 131.25, 129.79, 129.05, 127.12, 125.44, 121.06, 119.78, 117.80, 117.43, 115.99, 112.66, 74.08. **HRMS** (ESI+): *m/z* calculated for $C_{20}H_{12}N_2O_4$ $[M+H]^+$: 345.0797, found 345.0858.

2-Methyl-3*H*-chromeno[5',6':4,5]furo[2,3-*b*]pyridin-3-one (22) [3]. A mixture of **16** (0.1 g, 0.47 mmol), propionic anhydride (162 μL, 1.27 mmol), sodium propionate (99 mg, 1.0 mmol) and piperidine (46 μL, 0.5 mmol) was heated to reflux for 6 h. The mixture was then poured into 3 N HCl (5 mL) and stirred overnight. The solid was filtered off, washed with water and purified by column chromatography to yield **22** as white solid. (54 mg, 46%). **Mp** 240-242 °C; **^{1}H NMR** (400 MHz, $CDCl_3$) *δ*: 8.54 (dd, *J* = 4.9, 1.7, 1H), 8.46 (dd, *J* = 7.64, 1.7 Hz, 1H), 8.15 (s, 1H), 7.72 (d, *J* = 9 Hz, 1H), 7.48 (d, *J* = 9 Hz, 1H), 7.44 (dd, *J* = 7.7, 4.9 Hz, 1H), 2.37 (s, 1H). **^{13}C NMR** (100 MHz, $CDCl_3$) *δ*: 163.53, 161.73, 150.73, 150.13, 147.28, 134.86, 131.00, 127.82, 119.70, 117.66, 117.11, 116.31, 114.67, 114.45, 17.86. **HRMS** (ESI+): *m/z* calculated for $C_{15}H_9NO_3$ $[M+H]^+$: 252.0582, found 252.0640.

Ethyl 3-oxo-3*H*-chromeno[5',6':4,5]furo[2,3-*b*]pyridine-2-carboxylate (23) [4]. To a solution of diethyl malonate (78 μL, 0.52 mmol) and **16** (0.1 g, 0.47 mmol) in EtOH (3 mL) were added 3 drops of piperidine and an equal volume of acetic acid. The mixture was refluxed for 5 h. The reaction mixture was diluted with water and extracted with EtOAc (3 × 50 mL).

The combined organic layers were washed with brine and dried over $MgSO_4$. The solvent was removed under reduced pressure, and the crude reaction mixture was purified by column chromatography to obtain **23** as a yellow solid (90 mg, 62%) **Mp** >300 °C; **^{1}H NMR** (400 MHz, $CDCl_3$) *δ*: 9.15 (s, 1H), 8.60 (dd, *J* = 4.9, 1.7 Hz, 1H), 8.54 (dd, *J* = 7.8, 1.7 Hz, 1H), 7.93 (d, *J* = 9.0 Hz, 1H), 7.53 (d, *J* = 9.0 Hz, 1H), 7.50 (dd, *J* = 7.7, 4.9 Hz, 1H), 4.51 (q, *J* = 7.1 Hz, 2H), 1.47 (t, *J* = 7.1 Hz, 3H). **^{13}C NMR** (101 MHz, $CDCl_3$) *δ*: 163.69, 163.44, 156.33, 152.58, 150.73, 148.21, 144.19, 131.45, 120.15, 119.57, 119.33, 118.46, 117.12, 115.72, 62.60, 14.43. **HRMS** (ESI+): *m/z* calculated for $C_{17}H_{11}NO_5$ $[M+H]^+$: 310.0637, found 310.0703.

References

(1) Chang, J.; Zhao, K.; Pan, S. *Tetrahedron Lett.* **2002**, *43* (6), 951–954. doi:10.1016/S0040-4039(01)02302-4

(2) Vroemans, R.; Verhaegen, Y.; Dieu, M. T. T.; Dehaen, W. *Beilstein J. Org. Chem.* **2018**, *14*, 2689–2697. doi:10.3762/bjoc.14.246

(3) Gia, O.; Uriarte, E.; Zagotto, G.; Baccichetti, F.; Antonello, C.; Marciani-Magno, S. *J. Photochem. Photobiol. B Biol.* **1992**, *14* (1–2), 95–104. doi:10.1016/1011-1344(92)85085-9

(4) Oliveira, A. M. A. G.; Raposo, M. M. M.; Oliveira-Campos, A. M. F.; Griffiths, J.; Machado, A. E. H. *Helv. Chim. Acta* **2003**, *86* (8), 2900–2907. doi:10.1002/hlca.200390237

11 (^{1}H NMR, 400 MHz, $CDCl_3$)

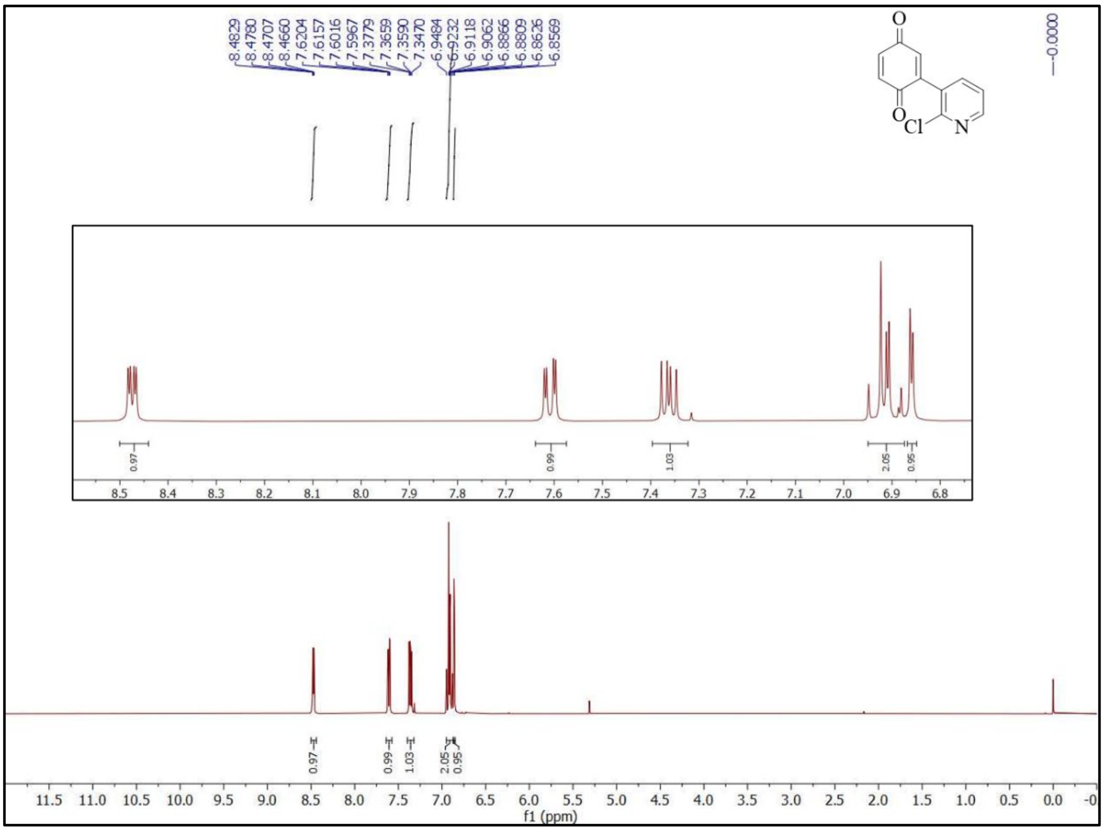

11 (^{13}C NMR, 101 MHz, $CDCl_3$)

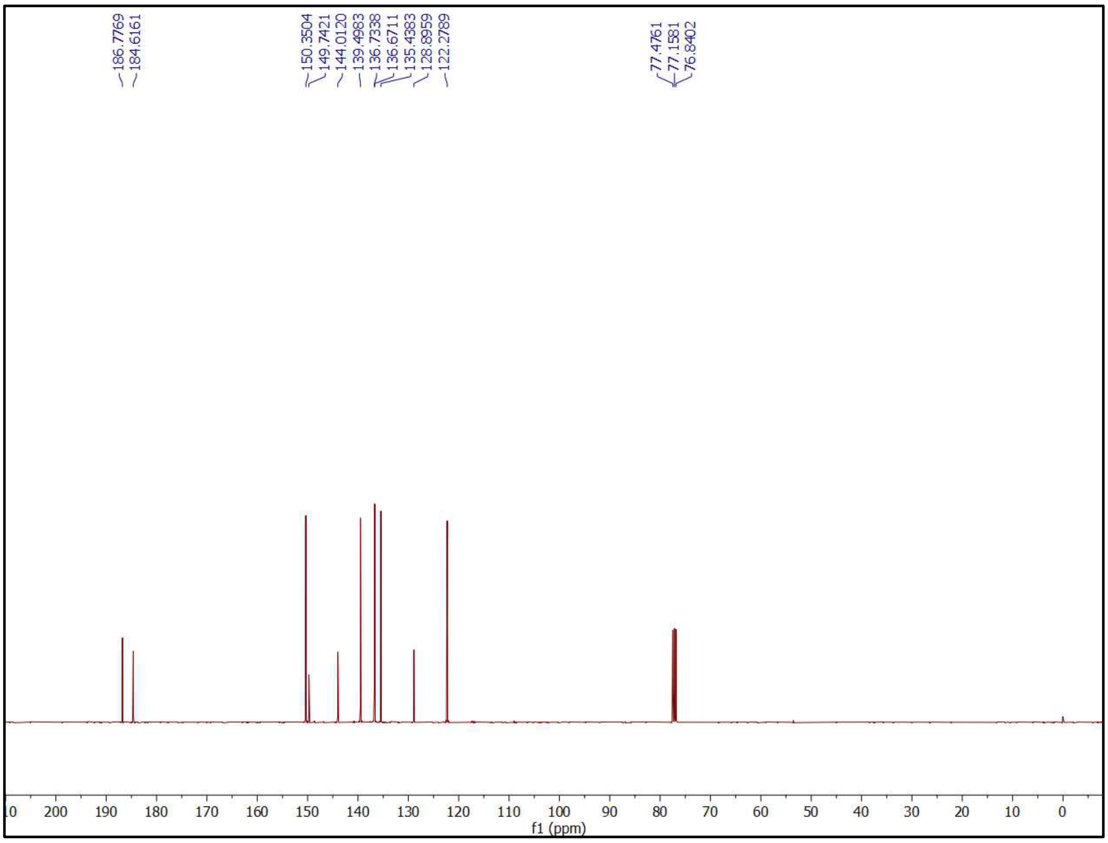

12 (^{1}H NMR, 400 MHz, DMSO-d_6)

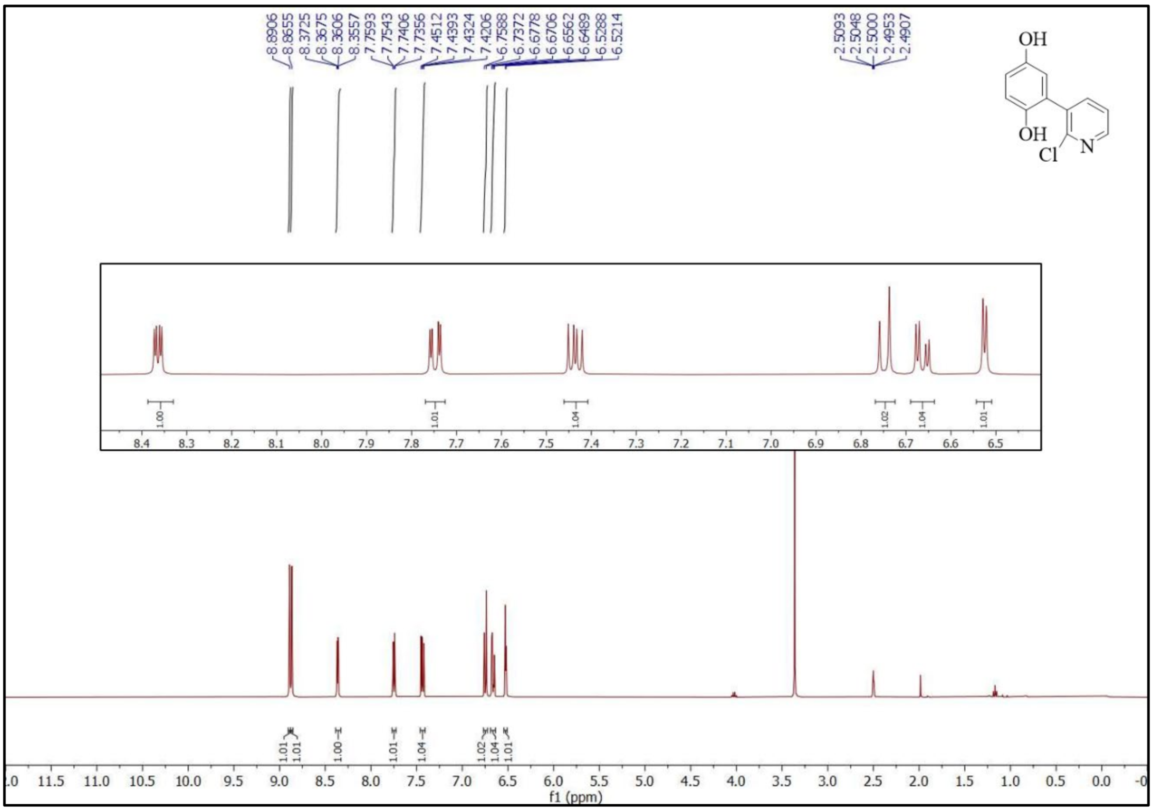

12 (^{13}C NMR, 101 MHz, DMSO-d_6)

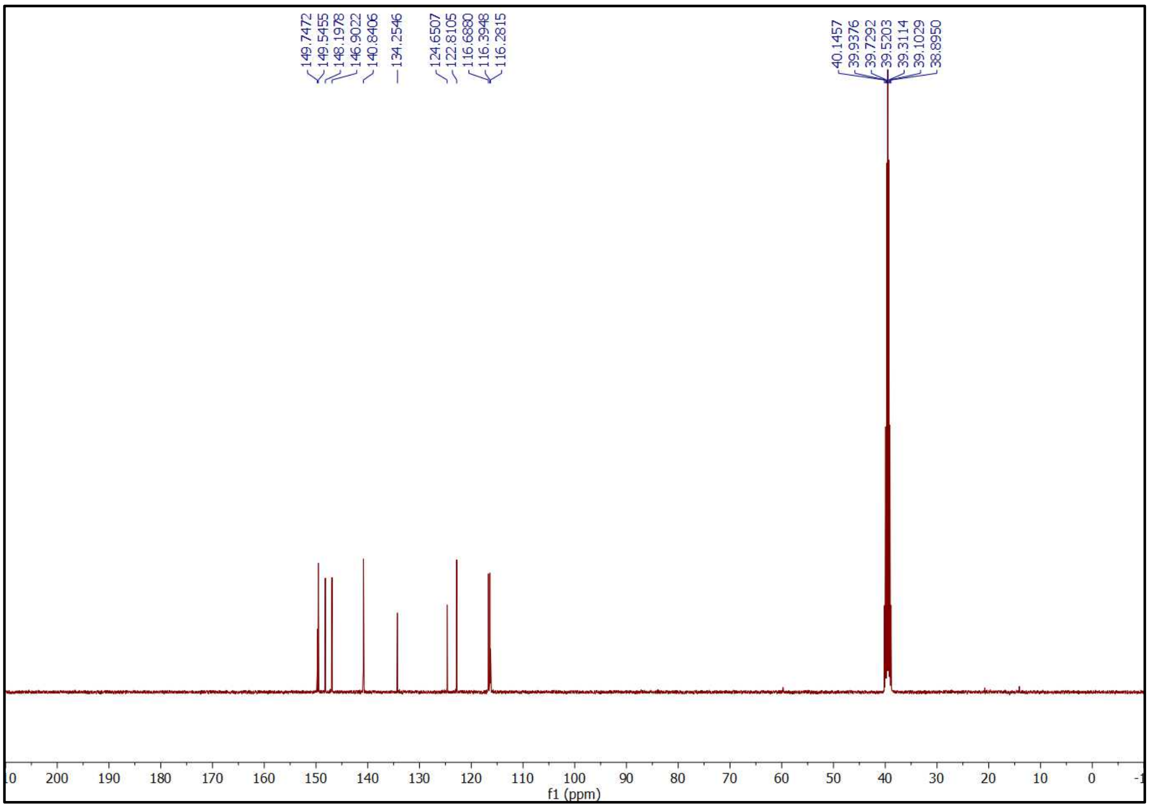

13 (^{1}H NMR, 400 MHz, DMSO-d$_6$)

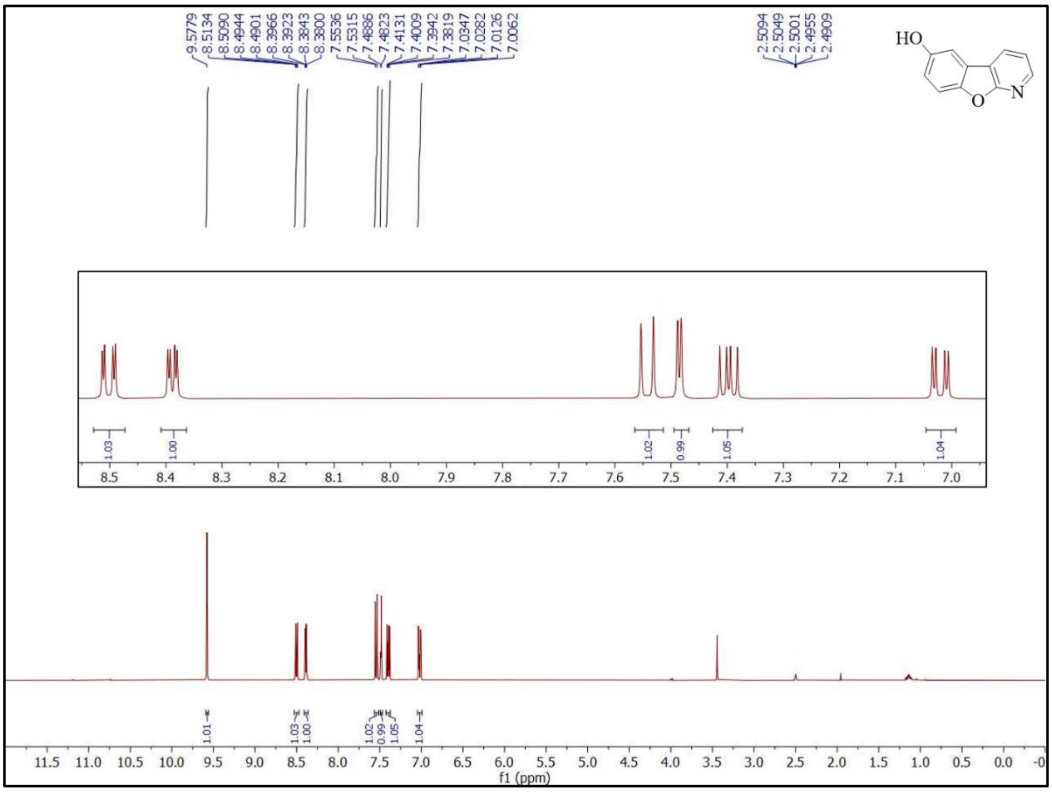

13 (^{13}C NMR, 101 MHz, DMSO-d_6)

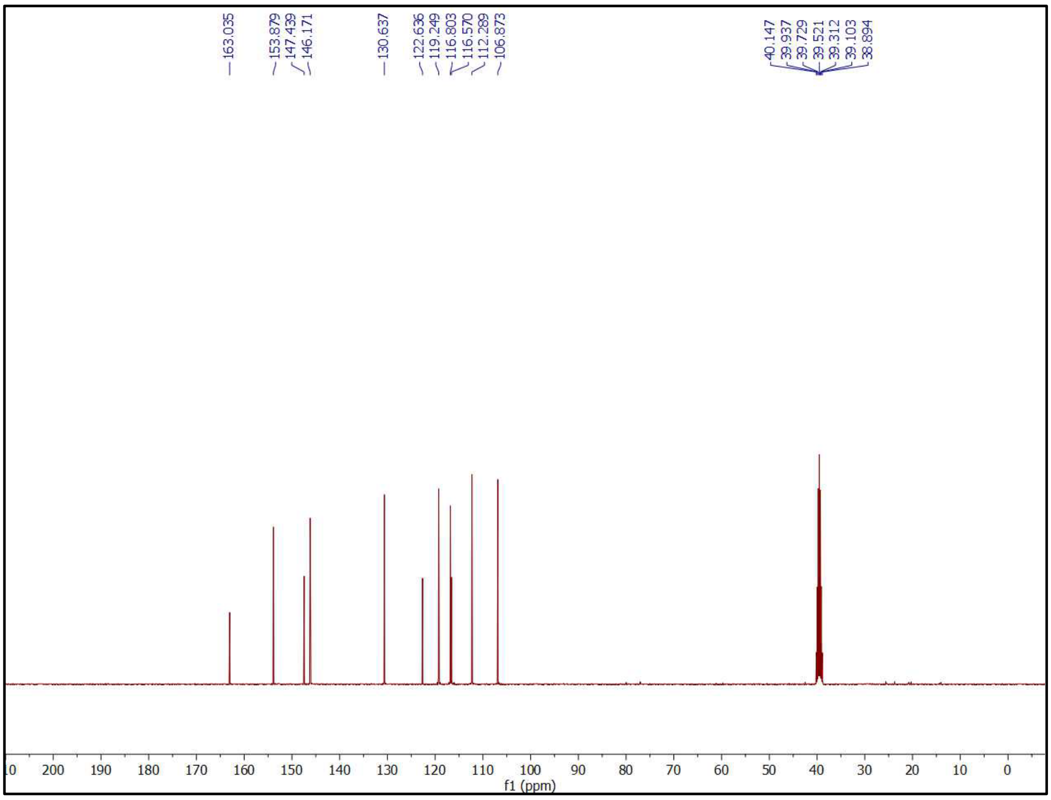

14 (^{1}H NMR, 400 MHz, DMSO-d_6)

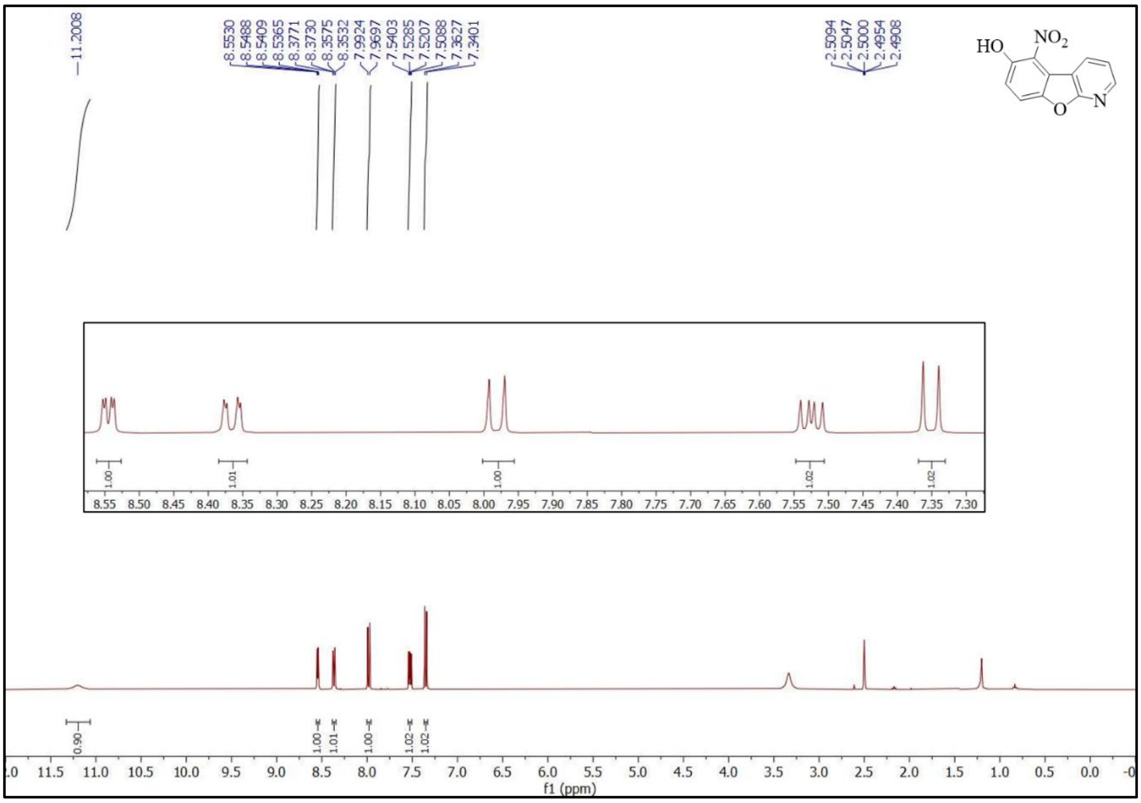

14 (^{13}C NMR, 101 MHz, DMSO-d_6)

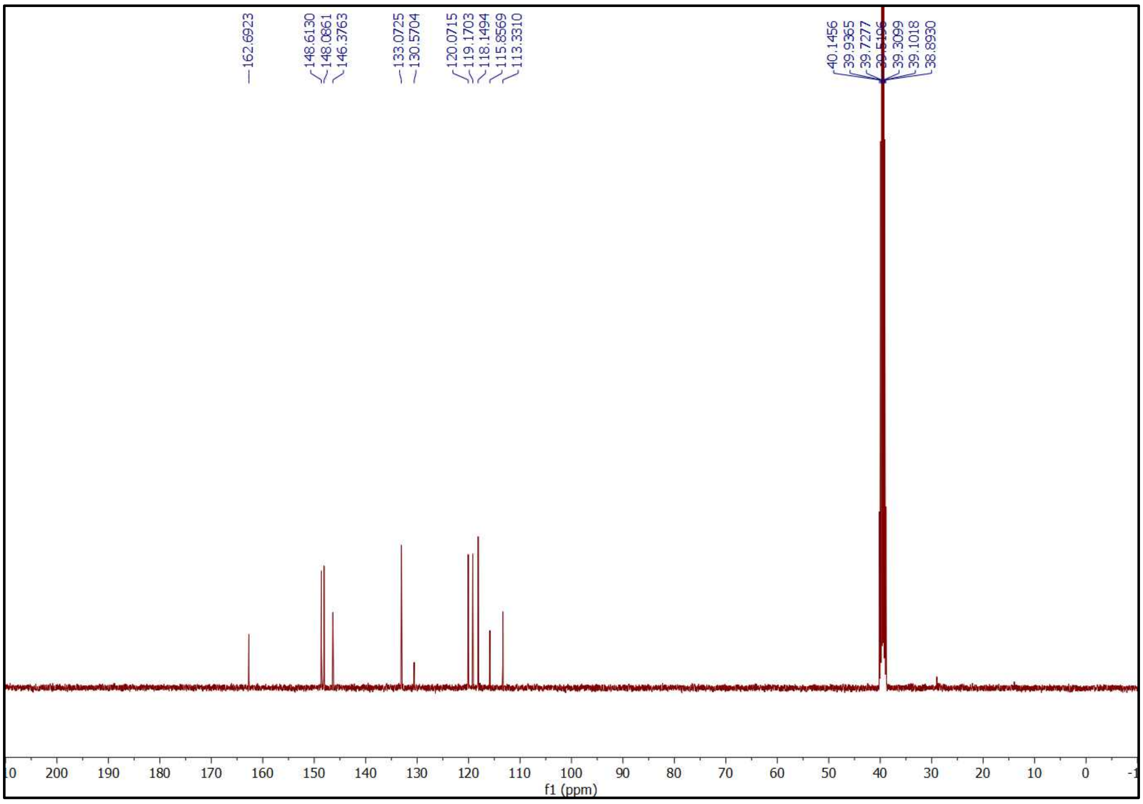

15 (^{1}H NMR, 400 MHz, DMSO-d_6)

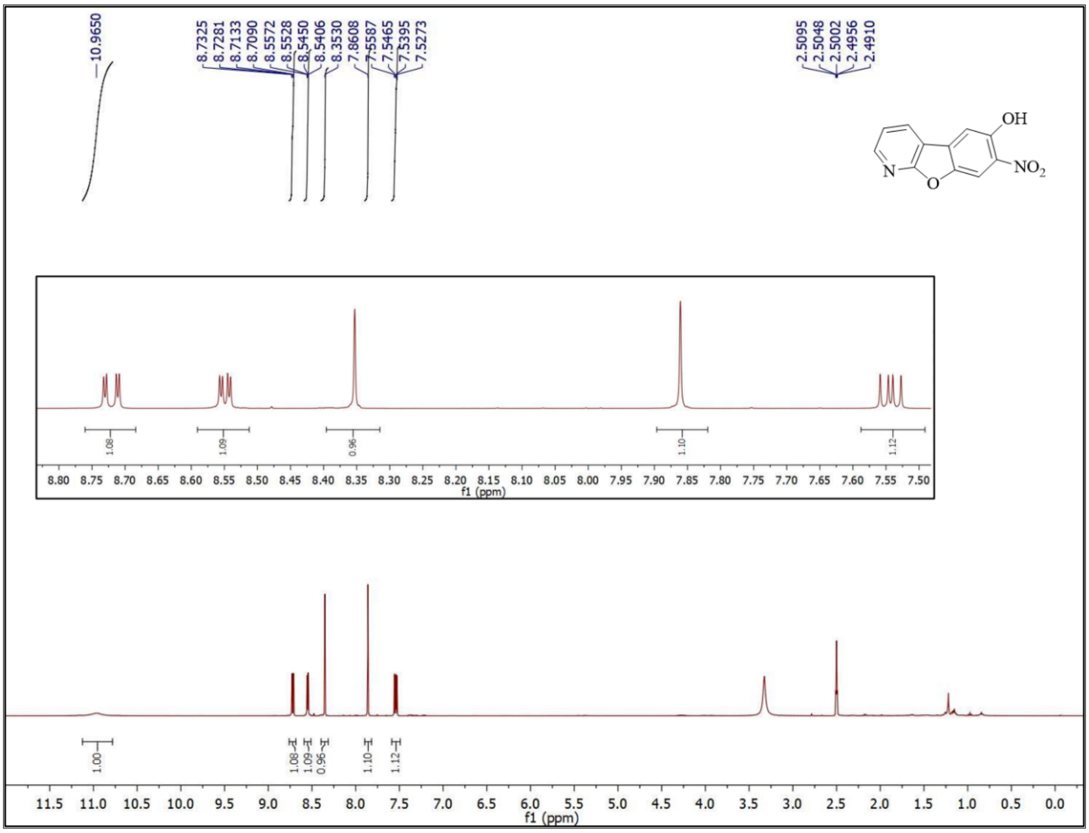

15 (^{13}C NMR, 101 MHz, DMSO-d_6)

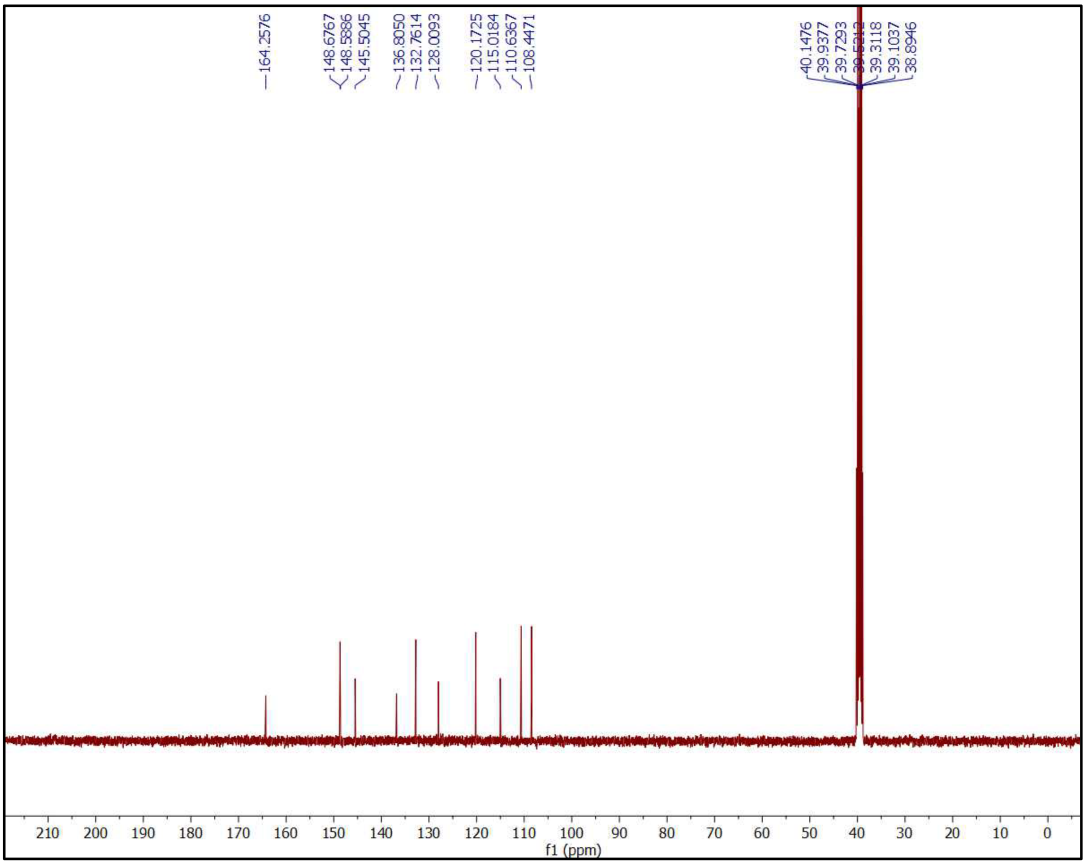

16 (^{1}H NMR, 400 MHz, DMSO-d_6)

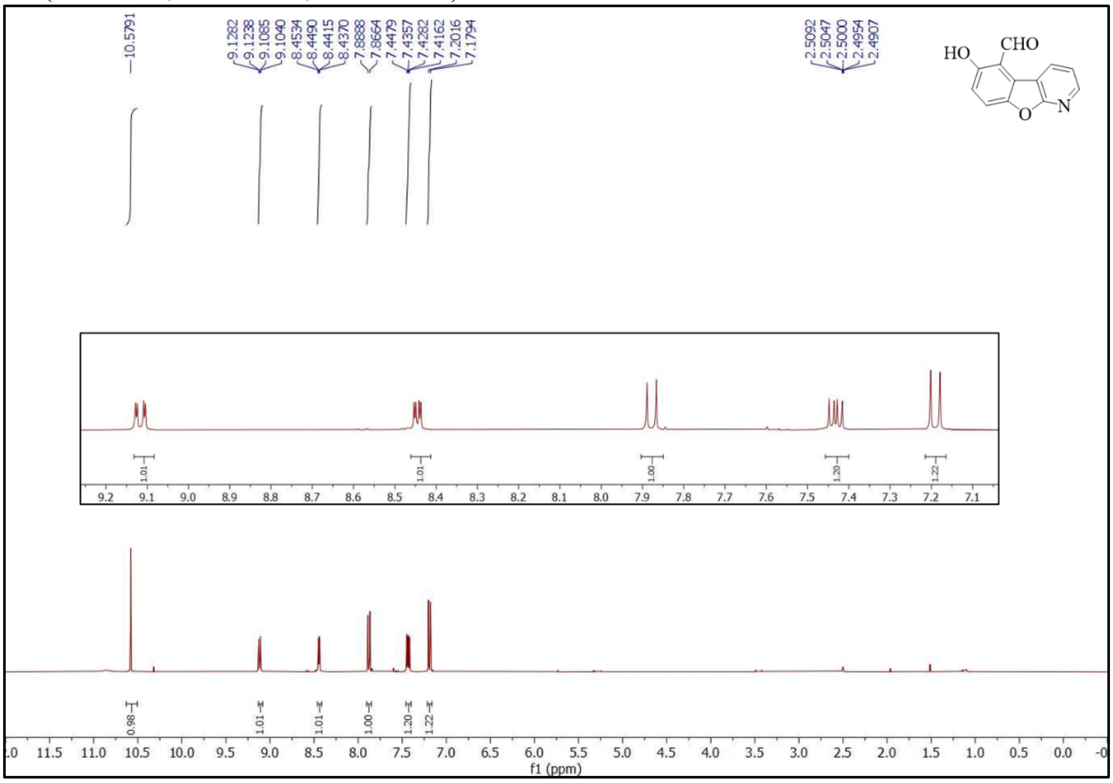

16 (^{13}C NMR, 101 MHz, DMSO-d_6)

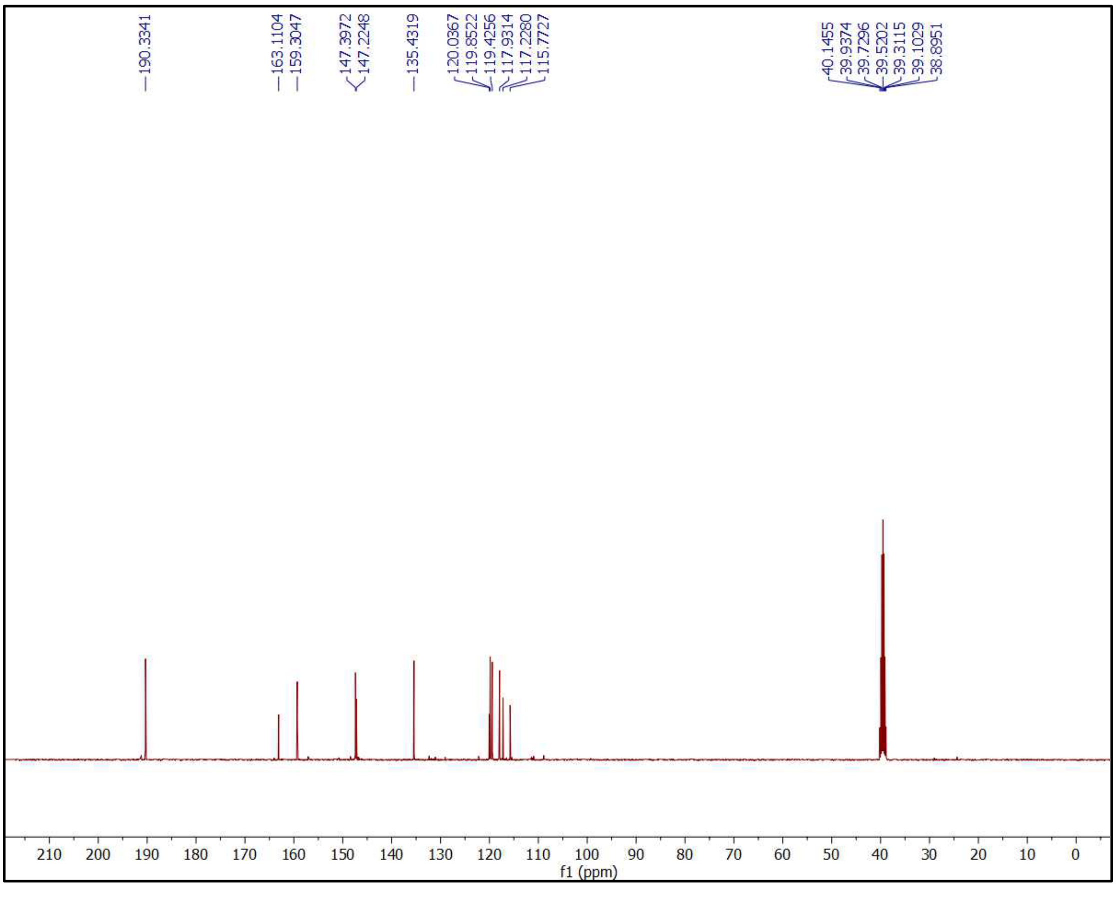

17 (^{1}H NMR, 400 MHz, DMSO-d_6)

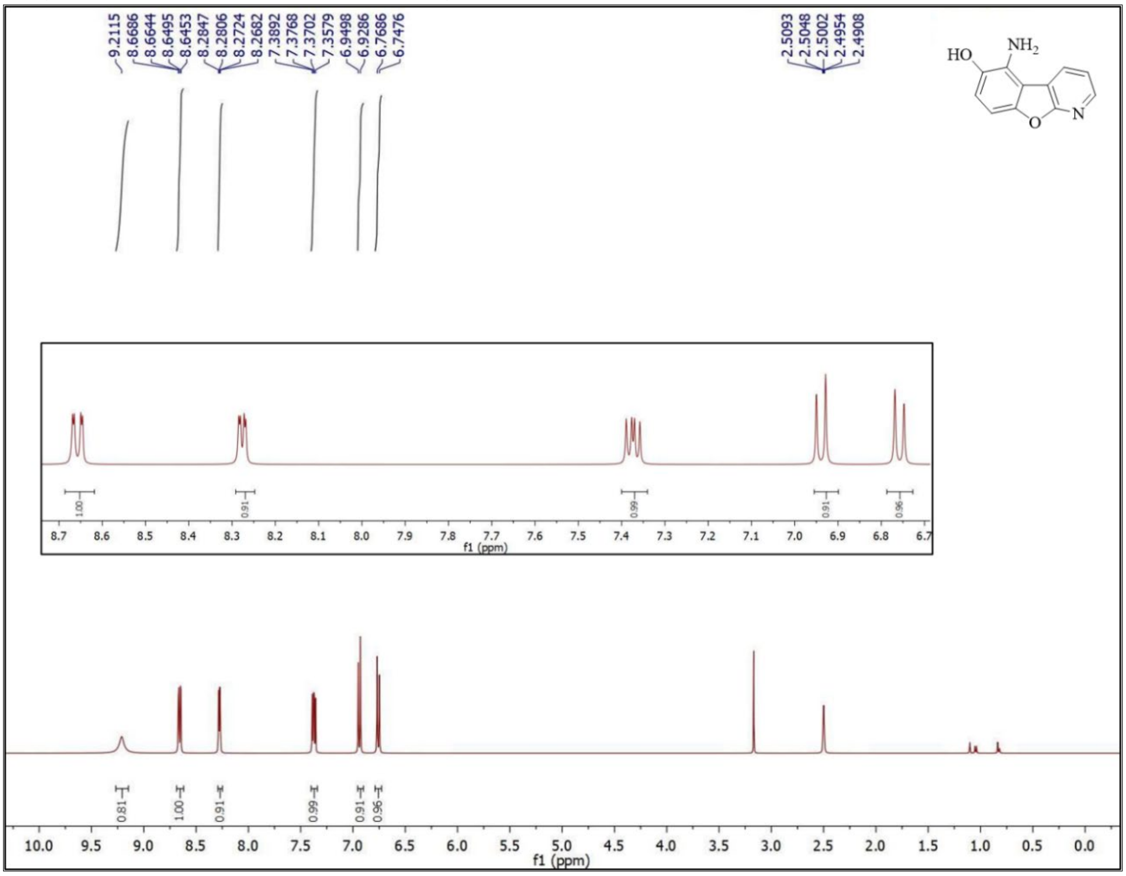

17 (^{13}C NMR, 101 MHz, DMSO-d_6)

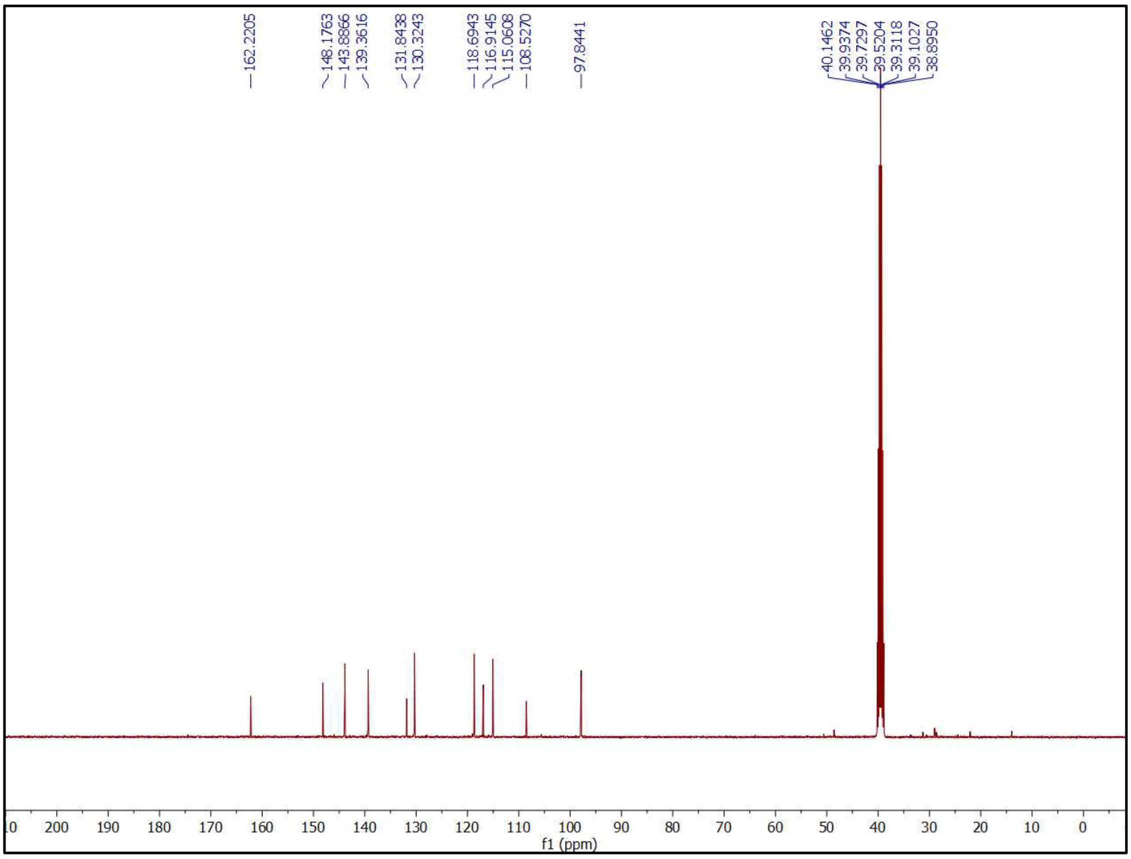

18 (^{1}H NMR, 400 MHz, DMSO-d_6)

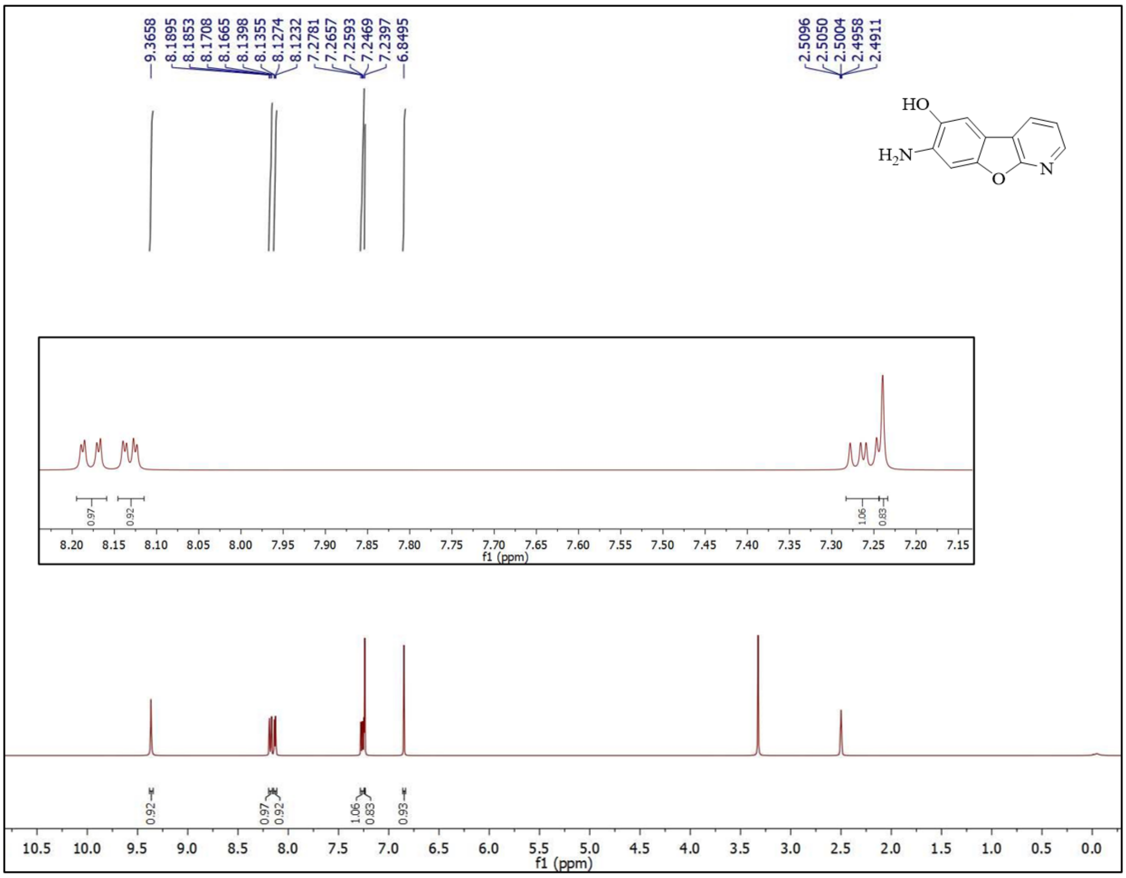

18 (^{13}C NMR, 101 MHz, DMSO-d_6)

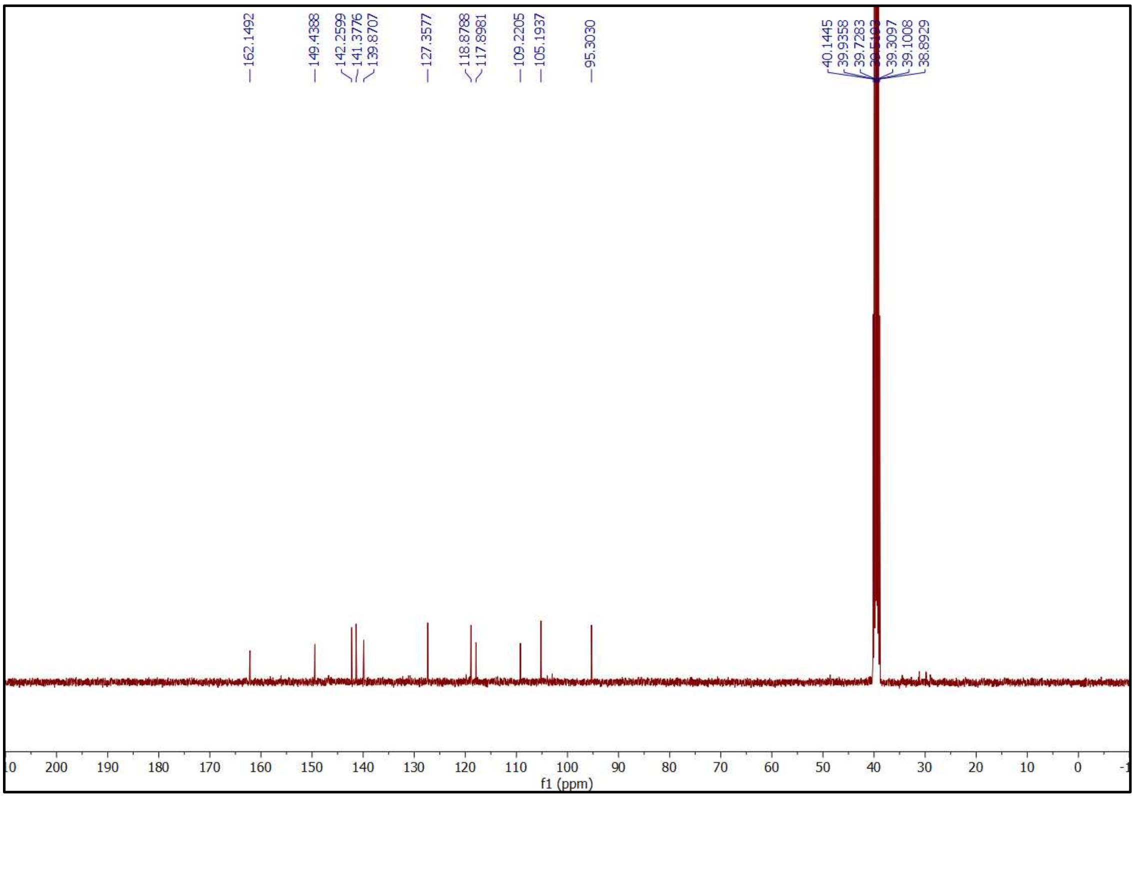

19 (^{1}H NMR, 400 MHz, DMSO-d_6)

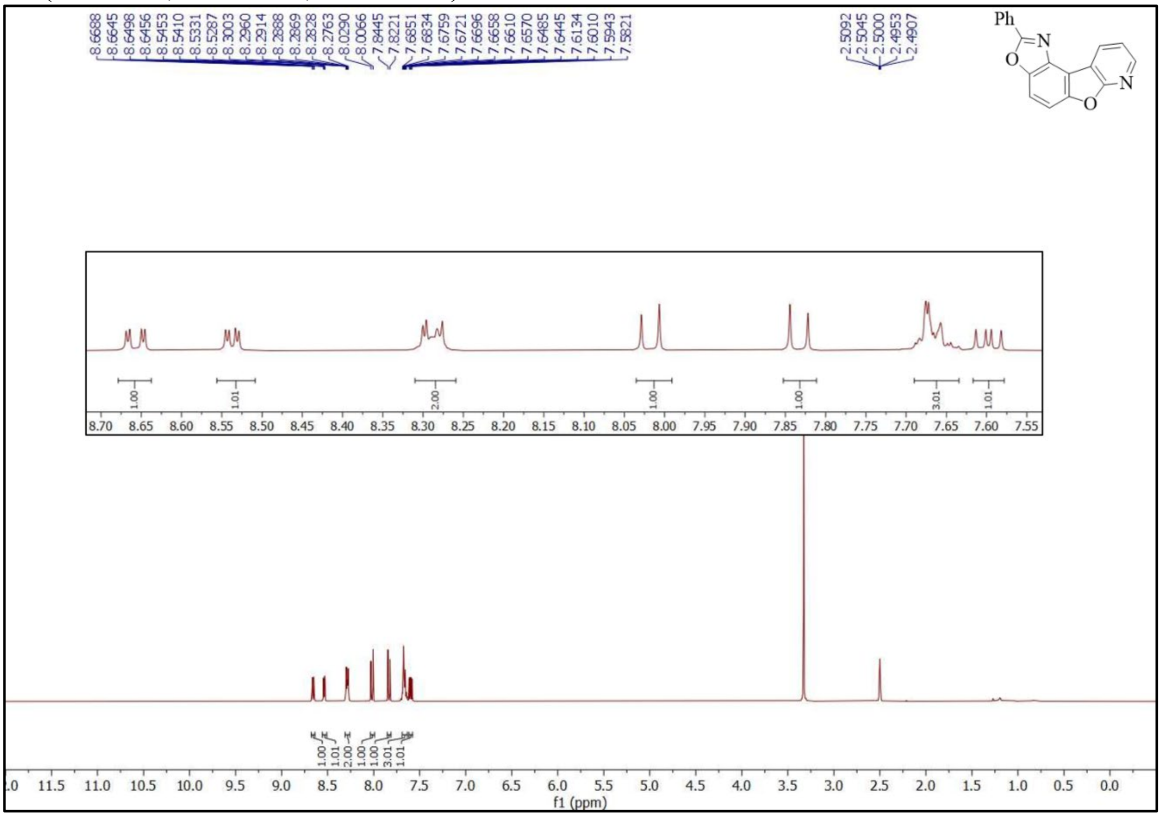

19 (^{13}C NMR, 101 MHz, DMSO-d_6)

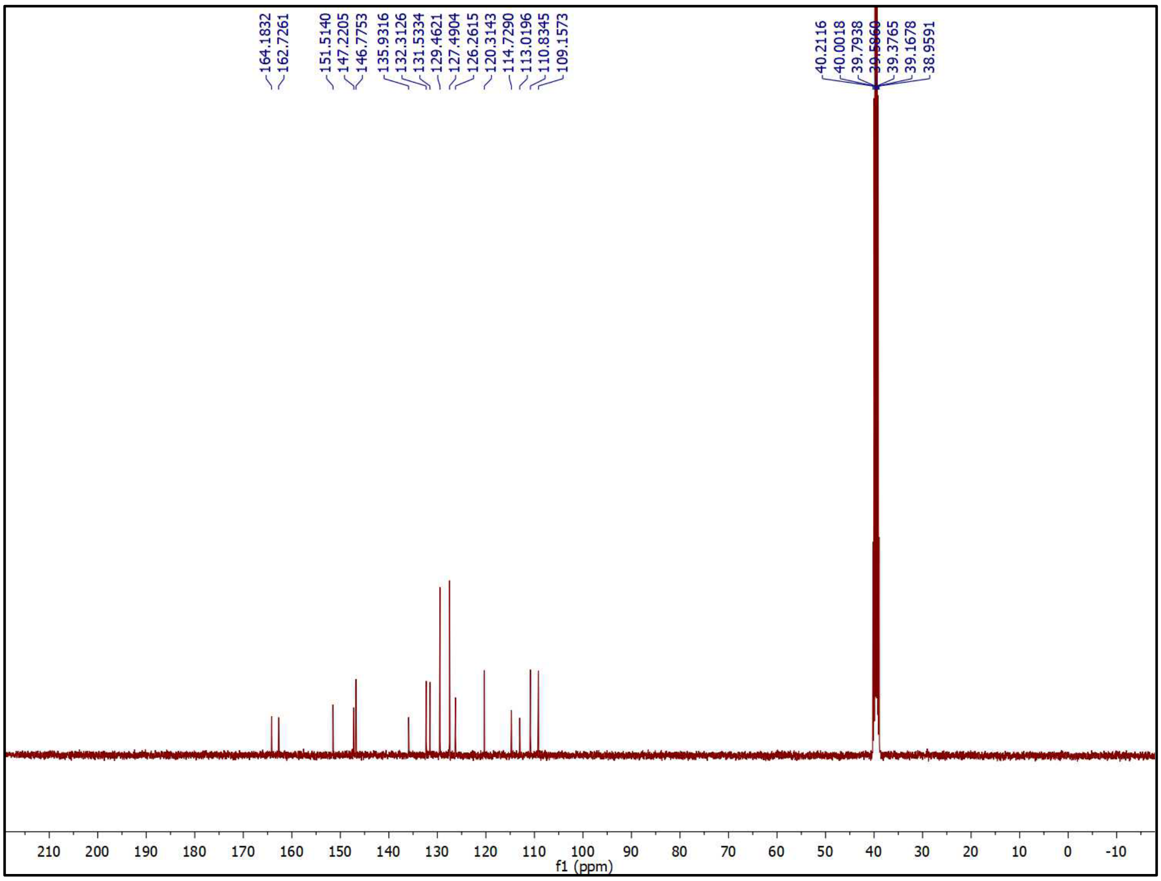

20 (^{1}H NMR, 400 MHz, $CDCl_3$)

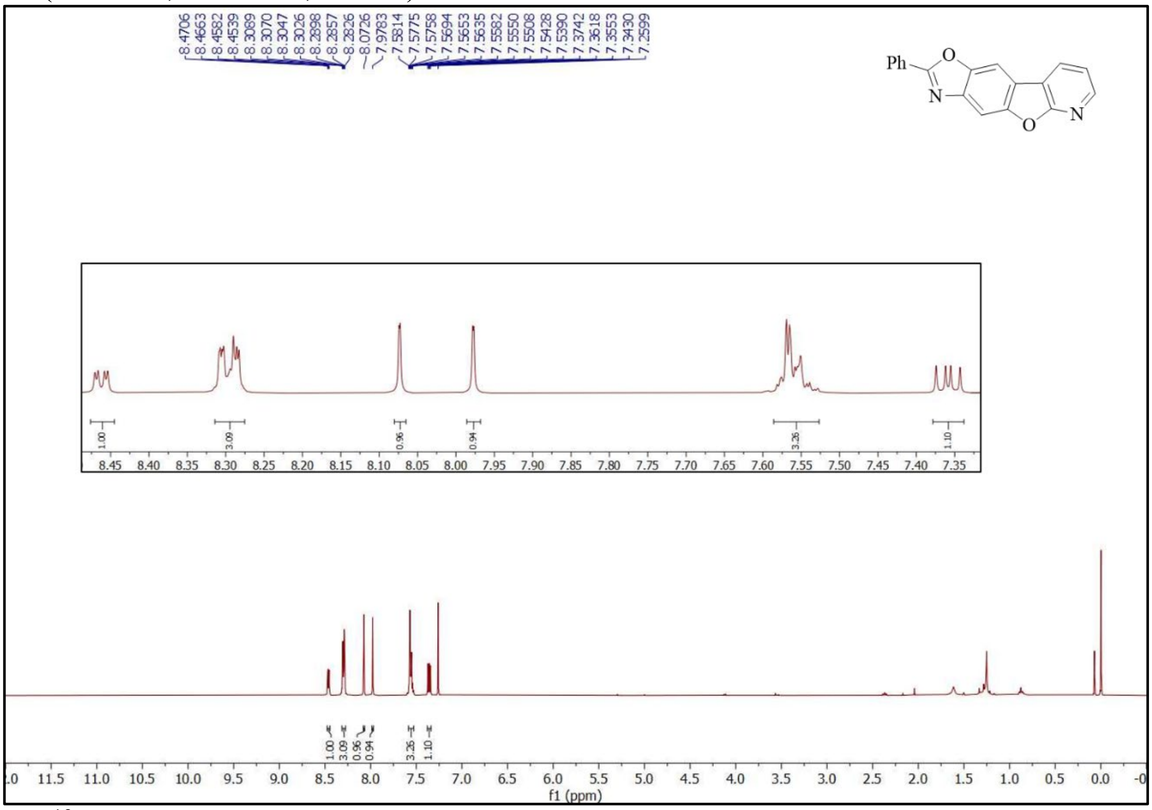

20 (^{13}C NMR, 101 MHz, $CDCl_3$)

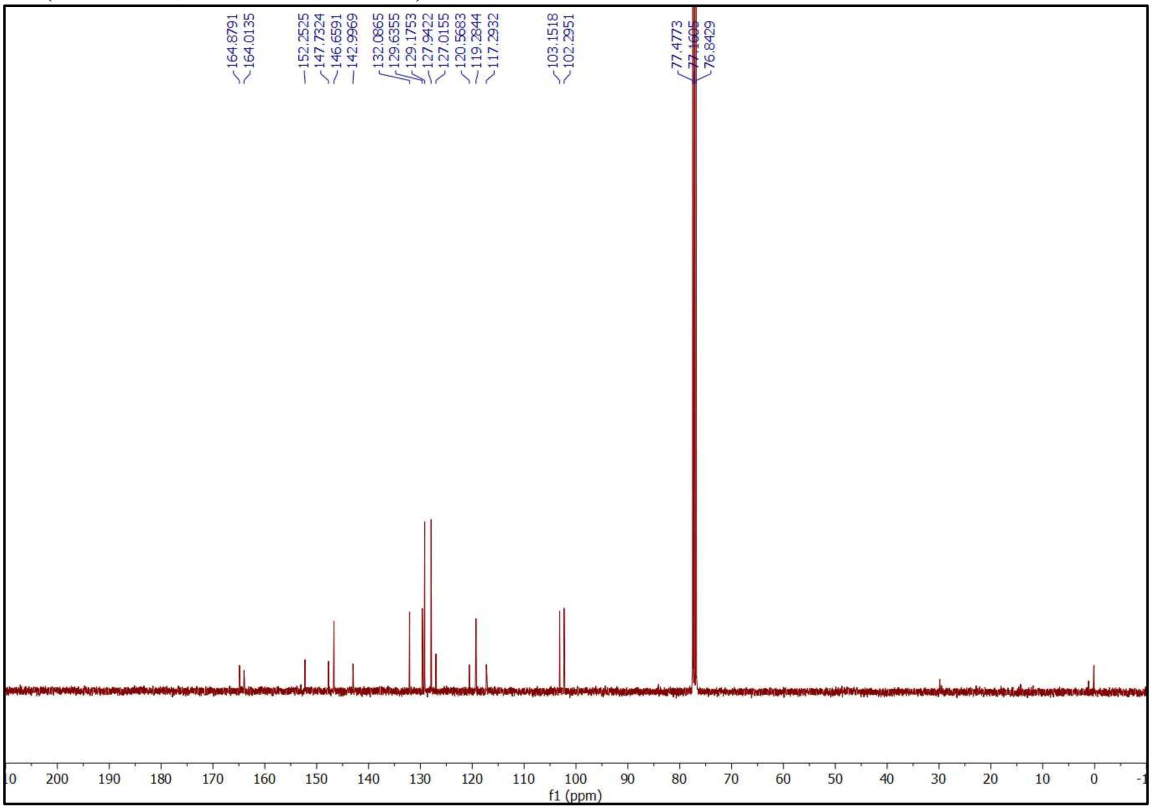

21 (^{1}H NMR, 400 MHz, $CDCl_3$)

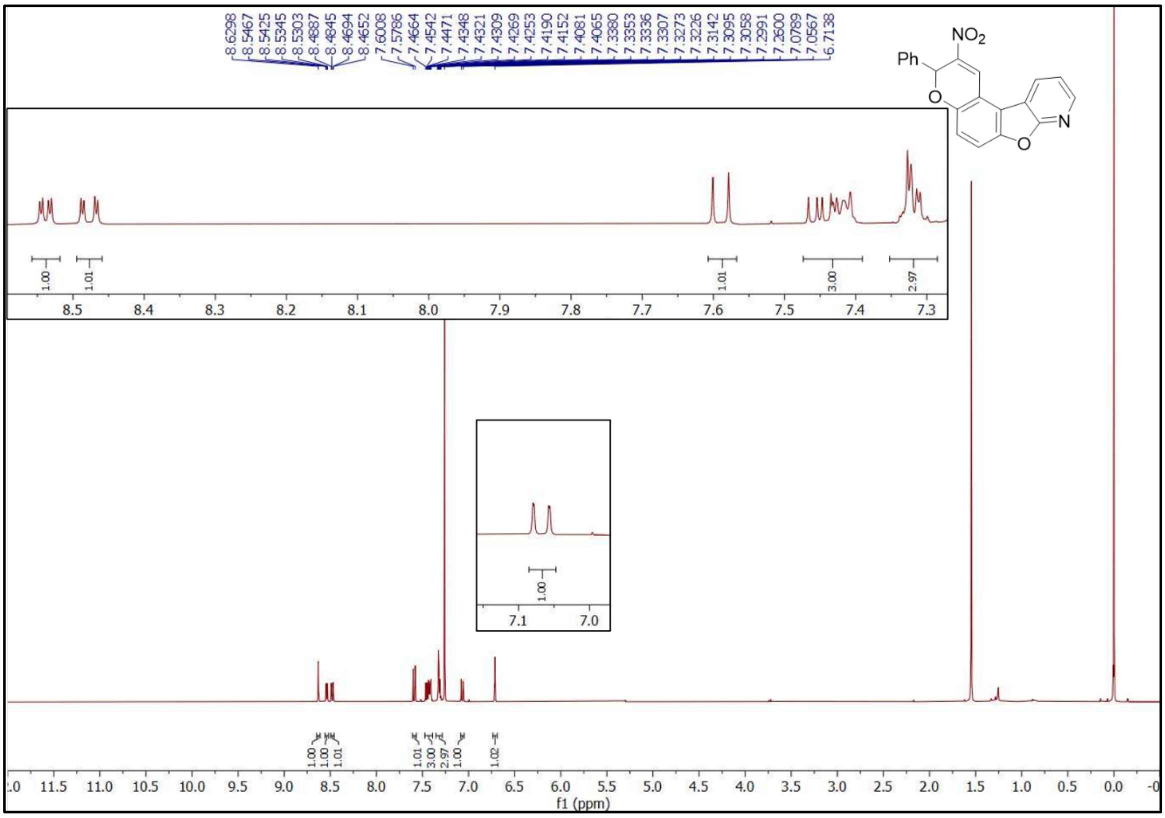

21 (^{13}C NMR, 150 MHz, $CDCl_3$)

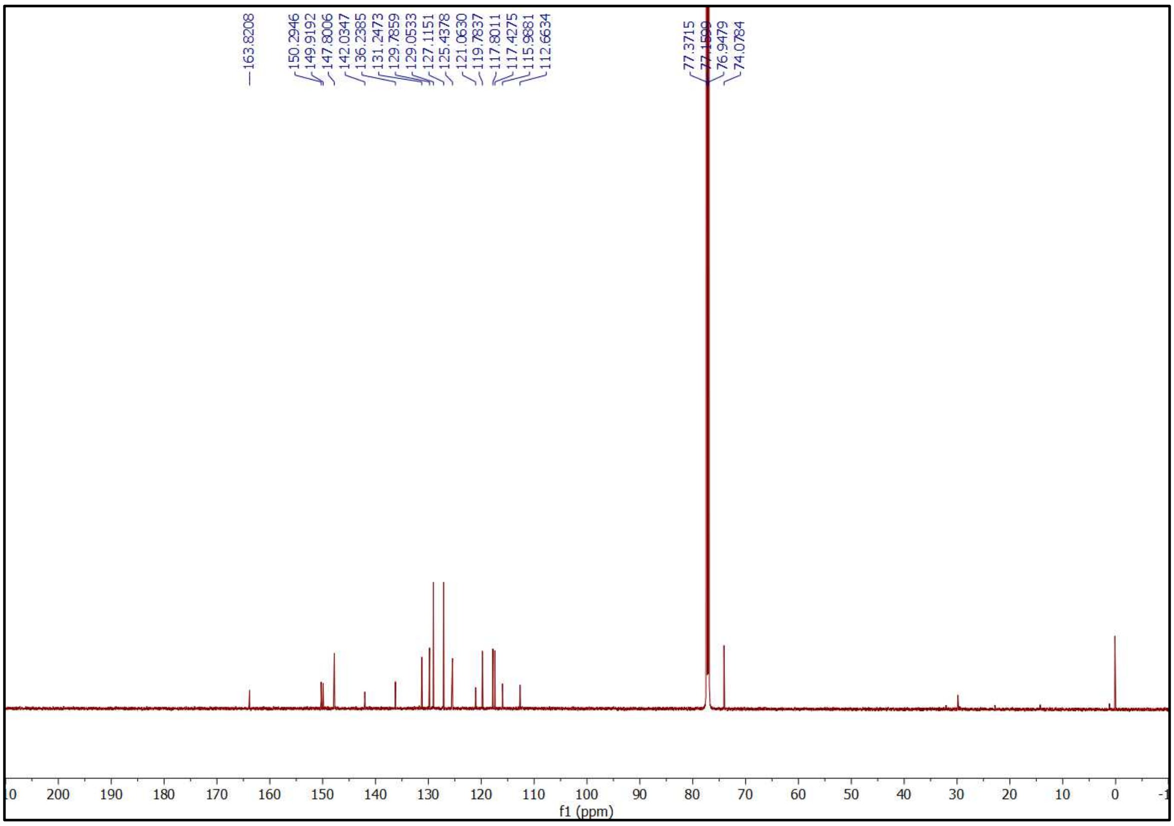

22 (^{1}H NMR, 400 MHz, $CDCl_3$)

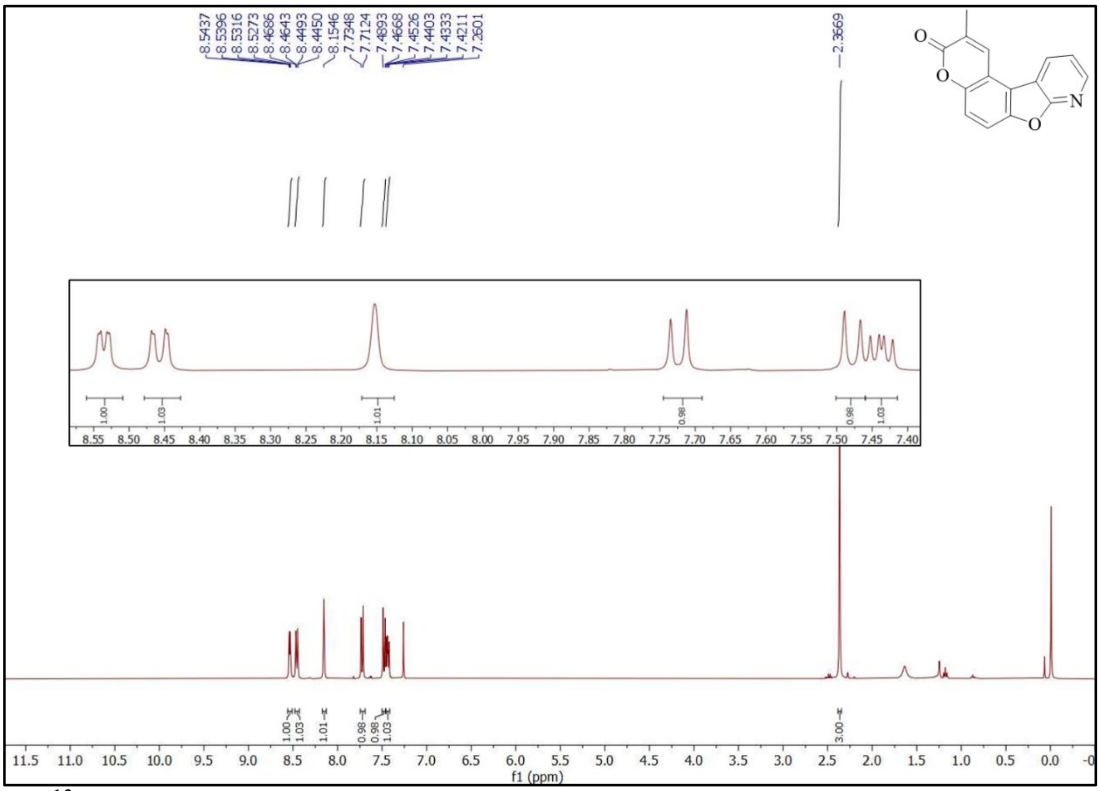

22 (^{13}C NMR, 101 MHz, $CDCl_3$)

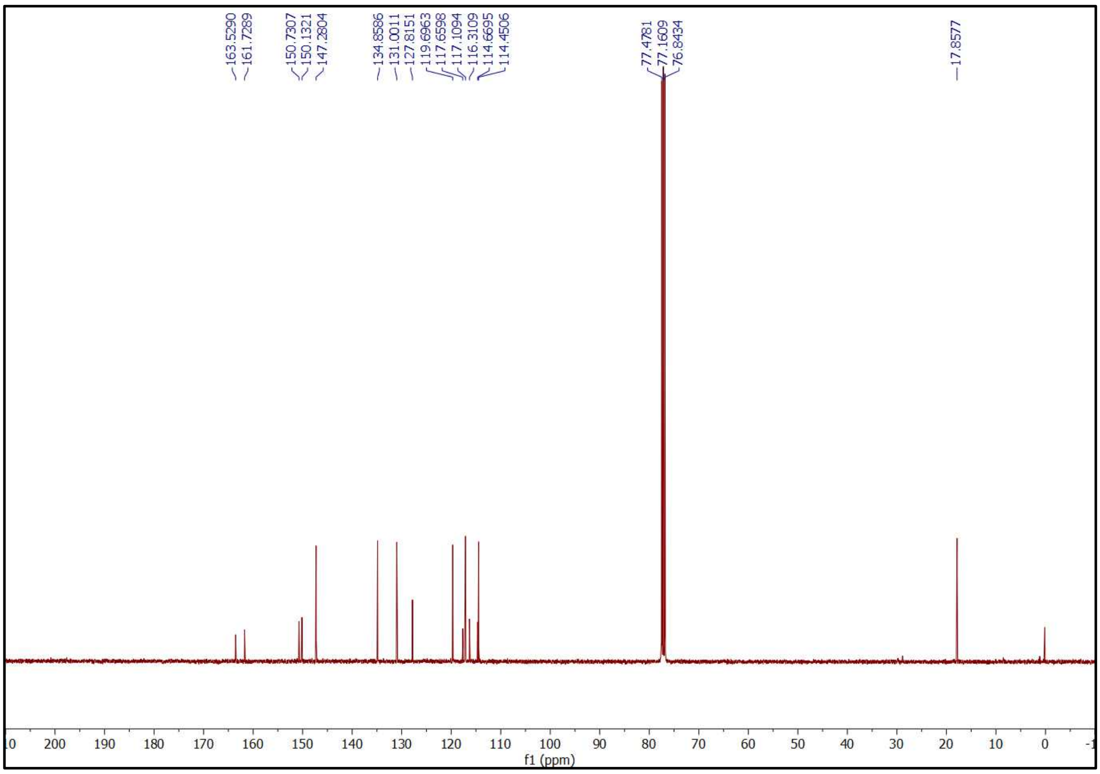

23 (^{1}H NMR, 400 MHz, $CDCl_3$)

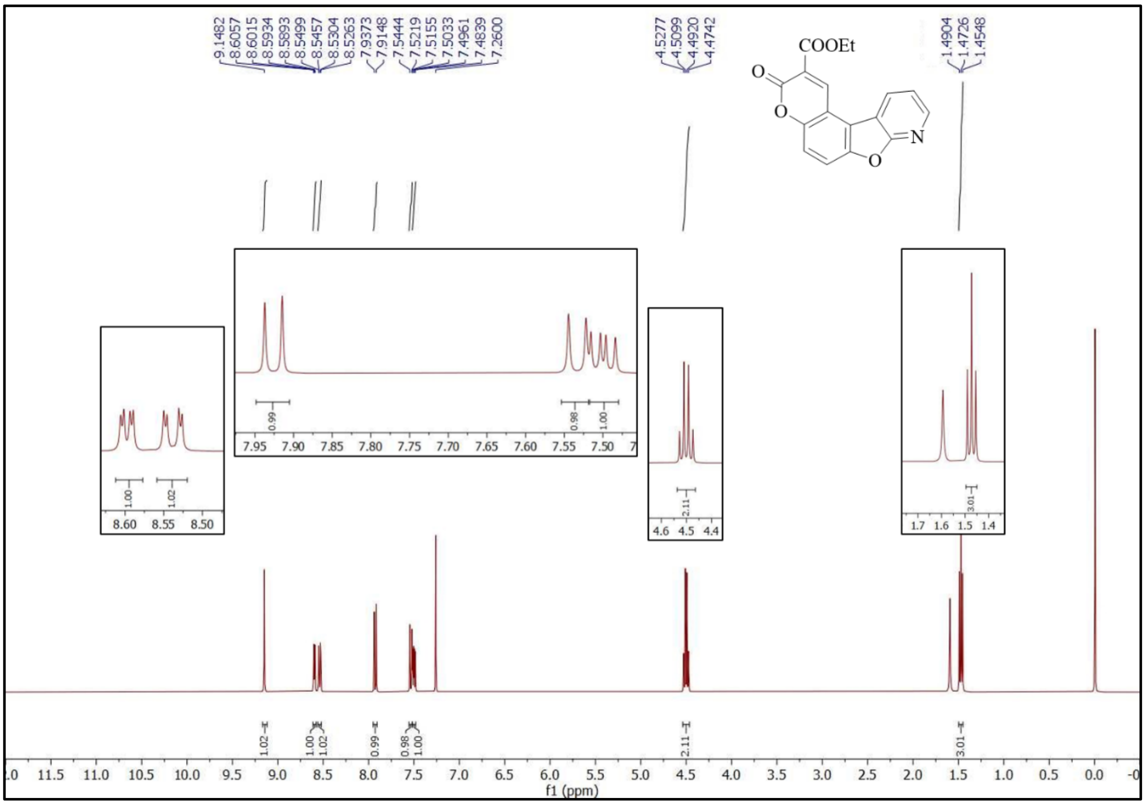

23 (^{13}C NMR, 101 MHz, $CDCl_3$)

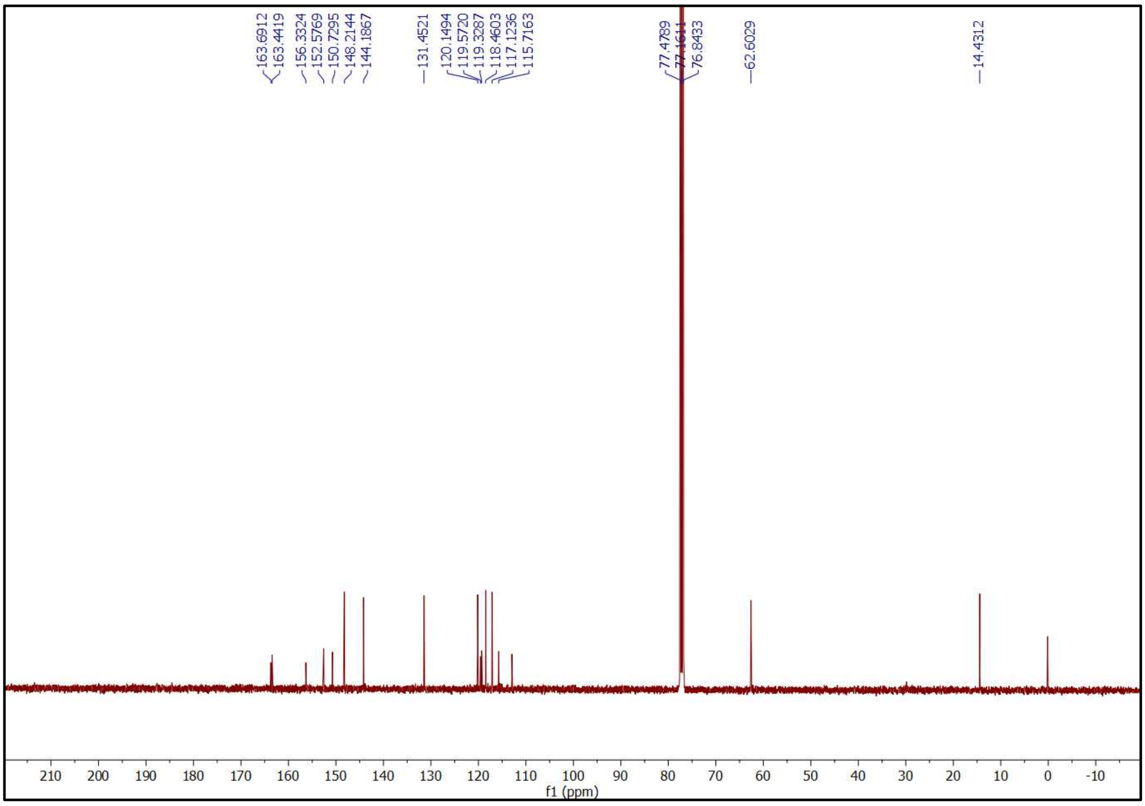

Supplement: File 1 — Experimental part as well as 1H and 13C NMR data. [file Beilstein_J_Org_Chem-17-977-s001.pdf]
